# Mechanical activation of spike fosters SARS-CoV-2 viral infection

**DOI:** 10.1038/s41422-021-00558-x

**Published:** 2021-08-31

**Authors:** Wei Hu, Yong Zhang, Panyu Fei, Tongtong Zhang, Danmei Yao, Yufei Gao, Jia Liu, Hui Chen, Qiao Lu, Tenny Mudianto, Xinrui Zhang, Chuxuan Xiao, Yang Ye, Qiming Sun, Jing Zhang, Qi Xie, Pei-Hui Wang, Jun Wang, Zhenhai Li, Jizhong Lou, Wei Chen

**Affiliations:** 1 Department of Cardiology of the Second Affiliated Hospital and Department of Cell Biology, Zhejiang University School of Medicine, Hangzhou, Zhejiang China; 2 Key Laboratory of RNA Biology, CAS Center for Excellence in Biomacromolecules, Institute of Biophysics, Chinese Academy of Sciences, Beijing, China; 3 School of Mechanical Engineering, Zhejiang University, Hangzhou, Zhejiang China; 4 Department of Hepatobiliary and Pancreatic Surgery, The Center for Integrated Oncology and Precision Medicine, Affiliated Hangzhou First People’s Hospital, Zhejiang University School of Medicine, Hangzhou, Zhejiang China; 5 Department of Pathology, New York University Grossman School of Medicine, New York, NY USA; 6 The Laura and Isaac Perlmutter Cancer Center, New York University Langone Health, New York, NY USA; 7 Collaborative Innovation Center for Diagnosis and Treatment of Infectious Diseases, the MOE Frontier Science Center for Brain Science & Brain-machine Integration, State Key Laboratory for Modern Optical Instrumentation Key Laboratory for Biomedical Engineering of the Ministry of Education, College of Biomedical Engineering and Instrument Science, Zhejiang University, Hangzhou, Zhejiang China; 8 Beijing National Laboratory for Condensed Matter Physics, Institute of Physics, Chinese Academy of Sciences, Beijing, China; 9 Advanced Medical Research Institute, Cheeloo College of Medicine, Shandong University, Jinan, Shandong China; 10 Westlake Institute for Advanced Study, School of Life Sciences, Westlake University, Hangzhou, Zhejiang China; 11 Shanghai Key Laboratory of Mechanics in Energy Engineering, Shanghai Institute of Applied Mathematics and Mechanics, School of Mechanics and Engineering Science, Shanghai University, Shanghai, China; 12 University of Chinese Academy of Sciences, Beijing, China; 13 Bioland Laboratory (Guangzhou Regenerative Medicine and Health Guangdong Laboratory), Guangzhou, Guangdong China; 14 Zhejiang Laboratory for Systems and Precision Medicine, Zhejiang University Medical Center, Hangzhou, Zhejiang China

**Keywords:** Molecular biology, Structural biology

## Abstract

The outbreak of SARS-CoV-2 (SARS2) has caused a global COVID-19 pandemic. The spike protein of SARS2 (SARS2-S) recognizes host receptors, including ACE2, to initiate viral entry in a complex biomechanical environment. Here, we reveal that tensile force, generated by bending of the host cell membrane, strengthens spike recognition of ACE2 and accelerates the detachment of spike’s S1 subunit from the S2 subunit to rapidly prime the viral fusion machinery. Mechanistically, such mechano-activation is fulfilled by force-induced opening and rotation of spike’s receptor-binding domain to prolong the bond lifetime of spike/ACE2 binding, up to 4 times longer than that of SARS-S binding with ACE2 under 10 pN force application, and subsequently by force-accelerated S1/S2 detachment which is up to ~10^3^ times faster than that in the no-force condition. Interestingly, the SARS2-S D614G mutant, a more infectious variant, shows 3-time stronger force-dependent ACE2 binding and 35-time faster force-induced S1/S2 detachment. We also reveal that an anti-S1/S2 non-RBD-blocking antibody that was derived from convalescent COVID-19 patients with potent neutralizing capability can reduce S1/S2 detachment by 3 × 10^6^ times under force. Our study sheds light on the mechano-chemistry of spike activation and on developing a non-RBD-blocking but S1/S2-locking therapeutic strategy to prevent SARS2 invasion.

## Introduction

A novel severe acute respiratory syndrome coronavirus 2 (SARS-CoV-2, referred to as SARS2 thereafter) causes the pandemic of the coronavirus diseases 2019 (COVID-19), posing a serious threat to public health worldwide.^1,2^ Although SARS2 and SARS share ~80% nucleotide identity in the whole genome sequences, SARS2 is more infectious and has infected a tremendously larger population worldwide (https://www.gisaid.org). However, the underlying molecular mechanism, especially the viral invasion into host cells, still remains elusive.

SARS2, as well as SARS, belongs to the beta coronavirus family and utilizes its spike protein to recognize host receptors (e.g., angiotensin-converting enzyme II receptor, ACE2)^3–6^ to invade host cells. The initial entry of SARS2 or SARS into the host cell occurs in two vital steps, receptor recognition by the spike protein and subsequent conformational changes of the spike to form fusion machinery.^7–9^ Both steps are respectively governed by two subunits of the spike, S1 and S2. Receptor-binding domain (RBD) in the S1 subunit is mainly responsible for ACE2 recognition, and the S2 subunit forms fusion machinery to target host-cell plasma membrane (PM) after S1/S2 detachment (Supplementary information, Fig. S1a).^7–12^ The sequences of SARS2- and SARS-RBDs are similar (Supplementary information, Fig. S1b), with highly conserved ACE2 contact residues.^13–15^ Minimal structural changes of SARS2 spike upon ACE2 binding seem not significant enough to trigger the detachment of tightly associated S1 and S2 subunits, for which additional factors might be required.

SARS2 and SARS primarily target the respiratory tract associated with complex mechanical cues.^16–19^ For instance, tensile force induced by membrane bending has been reported to be involved in cell–cell contact as well as in endocytosis.^20–23^ These two physiological processes are reminiscent of viral attachment onto and entry into host cells, leaving the role of membrane bending in viral invasion enigmatic. Similar to endocytosis, once a virion attaches to the epithelium layers of the lung airway, the bent epithelial cell membrane might exert tensile force on the single spike/ACE2 binding complex, which inevitably impacts spike/ACE2 binding and resultant viral host recognition, attachment, and invasion. Several recent studies have reported the structures of the SARS2-RBD with human ACE2 in the static force-free condition, merely demonstrating a similar contact interface to that of the SARS-RBD/ACE2 complex (Supplementary information, Fig. S1c). It has also been reported that SARS2 and SARS spikes or RBDs bind to ACE2 with similar binding affinities,^24–26^ which hardly explains SARS2’s higher contagiousness than SARS. Moreover, S1/S2 tight contact observed from spike structures^26–29^ and the observation that the majority of spikes on pre-fused SARS2 viruses are in pre-fusion state^30,31^ hardly support the spontaneous S1/S2 dissociation model that is proposed based on the recent observation of post-fusion S2 protein in purified full-length wild-type spikes.^27^ All of these raise questions whether and how tensile force regulates spike’s dissociation from ACE2 during viral invasion into host cells, whether the mechano-dependent binding differentiates SARS2 and SARS, and whether follow-up S1/S2 detachment also requires or is accelerated by tensile force.

Herein, by integrating multiple biophysical approaches, we demonstrate that SARS2 exploits mechanical force to enhance its spike recognition of ACE2 and subsequently accelerate S1/S2 detachment for effective invasion into host cells. SARS2 shows greater force-enhanced spike recognition of ACE2 than SARS, in good agreement with its higher infectivity. Such mechanical enhancement is very likely to be a universal regulatory mechanism for the invasion of other beta-coronaviruses. A D614G variation of SARS2 spike enhances force-dependent spike recognition of ACE2 and speeds up the follow-up S1/S2 detachment simultaneously. Moreover, we also identify an S1/S2-binding, non-RBD-blocking, and neutralizing antibody derived from convalescent COVID-19 patients that can unexpectedly restrain force-accelerated S1/S2 detachment.

## Results

### Theoretical estimation of the mechanical force exerted on single spike/ACE2 bond

**Fig. 1:**
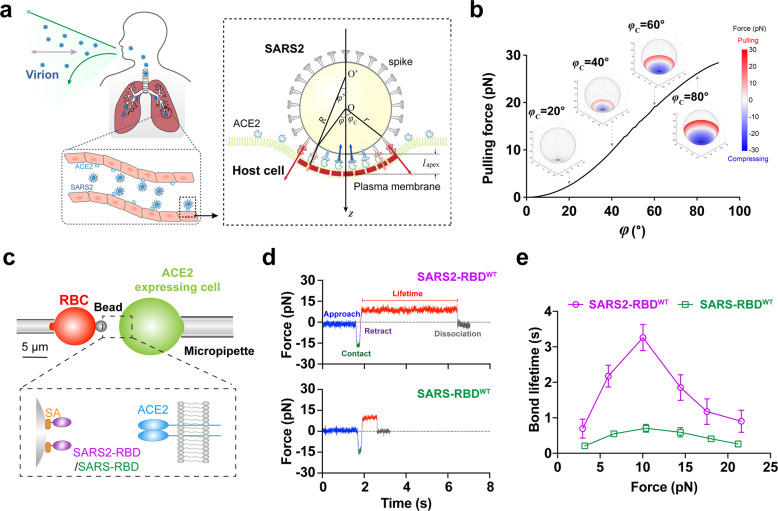
Mechanical force strengthens the binding between SARS2-RBD^WT^ or SARS-RBD^WT^ and ACE2. **a** Schematic diagram showing the mechano-environment of the SARS2 virus invading into human body through respiratory system. Host cell membrane is forced to bend by the spike/ACE2 interaction. **b** Theoretical estimation of the force exerted on single spike/ACE2 bond. Variation of the pulling force at the edge of the contact zone along with the *φ*_C_ change is shown (black curve). The distribution patterns of pulling and compressing forces when the contact zone grows to *φ*_C_ = 20°, 40°, 60° and 80° are shown in the insets. **c** Schematic diagram of biomembrane force probe setup and its functionalization strategy (zoomed-in panel). **d** Representative force vs time trace of the dissociation of SARS2-RBD^WT^ (upper panel) or SARS-RBD^WT^ (bottom panel) from ACE2 under force. Different phases are color-coded and indicated respectively. **e** Force-dependent bond lifetimes of SARS2-RBD^WT^/ACE2 or SARS-RBD^WT^/ACE2 binding. Error bars represent SEM.

Once a virion attaches to the host-cell PM through spike/ACE2 interaction, the contact zone gradually grows and enlarges, which is an energy-favored process. Upon spike/ACE2 binding, the potential energy of the virion/host-cell interaction system is reduced,^32^ and the released portion of the potential energy transfers to bend host-cell PM and deform spike/ACE2 bonds, thereby elevating the bending energy of host-cell PM and the elastic energy of deformed spike/ACE2 complexes.^23,33,34^ Driven by this energy conversion and owing to the softer host-cell PM compared to that of the virion,^35,36^ the host-cell PM inevitably bends to wrap the virion (Fig. 1a). Considering the mechanical equilibrium of both the virion and the host-cell PM, for a given contact zone (Fig. 1a, right), forces on the spike/ACE2 bonds within the contact zone between the virion and the host-cell can be calculated numerically (see Materials and Methods).

The force varies upon the contact zone growing (Supplementary information, Video S1), and the spike/ACE2 bonds in the center zone and the contact edge respectively bear compressing and pulling force (Fig. 1b, inset; Supplementary information, Video S1). The pulling force together with the compressing force maintains the bending of the PM, and the pulling force relies on and regulates the survival of the spike/ACE2 bonds. From our biomechanical analysis, the pulling force at the edge increases from 0 to 30 pN when the contact zone grows, and it reaches ~27 pN when *φ*_C_ is at 90° (Fig. 1b). In short, spike/ACE2 bonds should be subjected to tensile force during the SARS2 invasion, and the pulling force at the contact zone edge roughly ranges from 0 to 30 pN according to our theoretical analysis.

### Mechanical force prolongs SARS2-RBD/ACE2 bond lifetime to impede their dissociation

To test whether tensile force regulates spike/ACE2 binding and stiffness, we first carried out single-molecule biomechanical experiments with biomembrane force probe (BFP) to quantify the molecular stiffness of the spike/ACE2 complex (Supplementary information, Fig. S2) and the force-dependent RBD dissociation from ACE2 on live cells (Fig. 1c, d; Supplementary information, Fig. S3). We found that the molecular stiffness of the spike/ACE2 complex is about 1.8 pN/nm (Supplementary information, Fig. S2c). Increasing mechanical force at a low-force regime (< 10 pN) prolongs bond lifetimes of both SARS2-RBD^WT^ and SARS-RBD^WT^ binding with ACE2 (Fig. 1e; Supplementary information, Fig. S3c, d). Optimal force (~10 pN) results in maximum bond lifetimes (3.3 s and 0.7 s respectively for SARS2-RBD^WT^ and SARS-RBD^WT^ binding with ACE2), whereas further increasing force beyond 10 pN shortens their bond lifetimes. This optimal force falls in the theoretical range that each spike/ACE2 bond bears (Fig. 1b). This force-strengthened RBD/ACE2 binding suggests that mechanical cues can be exploited by both SARS2 and SARS to enhance its recognition of host ACE2 and attachment to host cells. Furthermore, the longer force-dependent bond lifetime of SARS2-RBD^WT^/ACE2 (Fig. 1e) is consistent with and might explain the higher infectivity of SARS2,^37^ despite similar contact areas in SARS2-RBD/ACE2 and SARS-RBD/ACE2 complex structures (Supplementary information, Fig. S1b, c) and comparable in-solution^24–26^ and in-situ binding affinities of SARS2-RBD or SARS-RBD to ACE2 (Supplementary information, Fig. S4). Together, these results suggest that the force-dependent dissociation rate of RBD/ACE2 binding is a key factor for regulating both SARS2 and SARS viral infection.

### Two force-induced intermediate binding states govern mechanical enhancement of SARS2-RBD/ACE2 interaction

**Fig. 2:**
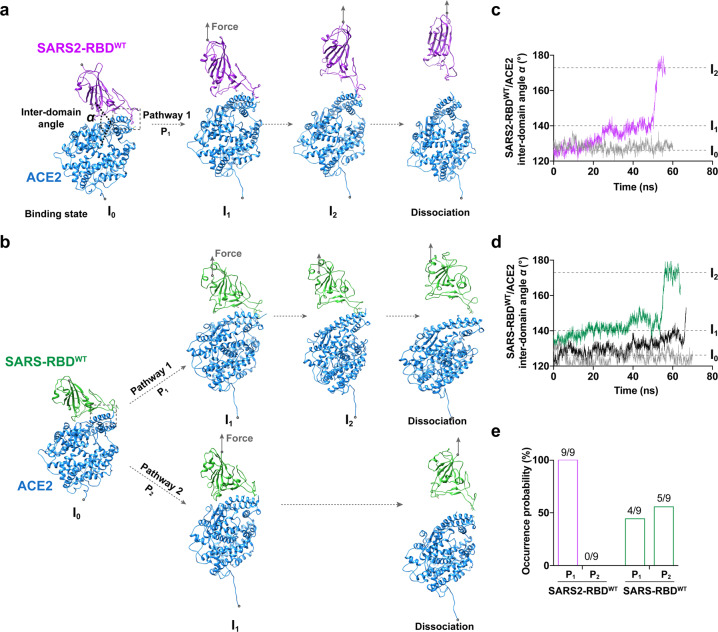
Force-dependent dynamical and structural mechanisms of the dissociation of SARS2-RBD^WT^ or SARS-RBD^WT^ from ACE2. **a**, **b** Sequential SMD snapshots of force-dependent SARS2-RBD^WT^ (**a**) and SARS-RBD^WT^ (**b**) dissociation from ACE2. SARS2-RBD^WT^/ACE2 dissociation adopts a sole pathway P_1_ with two intermediate states (I_1_ and I_2_), but SARS-RBD^WT^/ACE2 dissociation adopts two different dissociation pathways (P_1_ and P_2_), including two (I_1_ and I_2_) or one (only I_1_) intermediate state, respectively. I_0_ refers to the no-force state. Inter-domain angle (*α*) between RBD and ACE2, anchoring and force pulling residues (gray balls) and force directions (gray arrows) are indicated. **c**, **d** Representative time-courses of the inter-domain angle (*α*) between SARS2-RBD^WT^ (**c**) or SARS-RBD^WT^ (**d**) and ACE2 in the presence (purple in **c**; green (for P_1_) and black (for P_2_) in **d**) or absence (gray) of force. Horizontal dashed lines indicate the inter-domain angles in states of I_0_, I_1_, and I_2_. **e** The occurrence probabilities of P1 and P2 pathways in force-dependent dissociation of SARS2-RBD^WT^ or SARS-RBD^WT^ from ACE2.

To dissect the dynamical and structural mechanisms of the mechano-enhanced RBD/ACE2 binding, we next performed steered molecular dynamics (SMD) simulations on SARS2-RBD^WT^/ACE2 and SARS-RBD^WT^/ACE2 complexes, and examined their force-induced conformational change and dissociation pathway at atomic resolution. For SARS2-RBD^WT^/ACE2 dissociation, we found that the tensile force drove the SARS2-RBD^WT^ rotation on the binding interface and gradually increased the inter-domain angle (*α*) from ~125° at force-free initial state (I_0_) to ~140° (Intermediate state 1, I_1_) and then to ~170° (Intermediate state 2, I_2_) followed by RBD/ACE2 dissociation, and the inter-domain area (nm^2^) gradually decreased under the tensile force (Fig. 2a, c; Supplementary information, Fig. S5a, b, Video S2). In the I_1_ state, the binding conformation only changes very little compared with the force-free I_0_ state. In the I_2_ state, only RBD’s receptor-binding motif (RBM) interacts with ACE2 while the other regions dissociate (Supplementary information, Fig. S5c, e, f). Similar force-induced conformational changes on SARS2-RBD^WT^/ACE2 binding interface were observed along this dissociation pathway (denoted as P_1_) in all 9 independent simulations (Fig. 2e). Interestingly, other than the P_1_ pathway (Supplementary information, Video S3), another pathway (denoted as P_2_; Supplementary information, Video S4) was also identified (Fig. 2b, d; Supplementary information, Fig. S5a, b, d) in SARS-RBD^WT^/ACE2 dissociation. In the P_2_ pathway, the intermediate state I_2_ is absent, causing direct dissociation of SARS-2 RBD^WT^/ACE2 from the state I_1_. The P_1_ and P_2_ pathways occurred 4 and 5 times respectively in 9 independent simulations of SARS-RBD^WT^/ACE2 dissociation (Fig. 2e). Thus, the incidence of the P_2_ pathway in SARS-RBD^WT^/ACE2 forced dissociation is 55%, which is much higher than that in SARS2-RBD^WT^/ACE2 forced dissociation (0%, i.e., no occurrence). The ability of SARS-RBD to resist forced dissociation from ACE2 is weaker in the P_2_ than in the P_1_ pathway, as no force-induced drastic rotational conformational changes solely sustained by RBM/ACE2 binding occurred in the P_2_ pathway. Together, these findings provide biophysical evidence to support the force-strengthened bond lifetime of SARS2-RBD^WT^/ACE2 binding. We thus postulated that force-induced intermediate states governed dissociation pathway selection and force-enhanced SARS2-RBD^WT^/ACE2 binding.

**Fig. 3:**
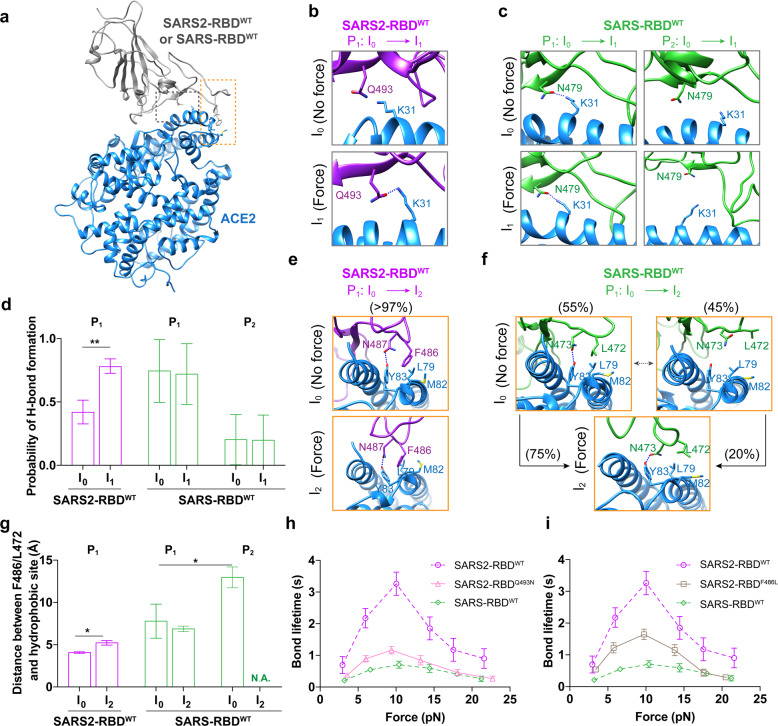
Identification of essential residues in SARS2-RBD^WT^ responsible for force-prolonged RBD/ACE2 bond lifetime. **a** Structure of the RBD/ACE2 complex with orange and gray dashed boxes highlighting residues involved in the force-regulated RBD/ACE2 interaction identified in SMD simulations. **b**–**d** Representative snapshots showing force-enhanced interaction network (zoomed-in view of the gray dashed box region in **a**) in the vicinity of indicated residues of SARS2-RBD^WT^ (**b**) or SARS-RBD^WT^ (**c**) in force-free state I_0_ or force-induced intermediate state I_1_. Their respective probabilities of H-bond formation are compared in **d**. **e**–**g** Representative snapshots showing interaction network (zoomed-in view of the orange dashed box region in **a**) in the vicinity of indicated residues of SARS2-RBD^WT^ (**e**) or SARS-RBD^WT^ (**f**) and ACE2 hydrophobic center in force-free state I_0_ or force-induced intermediate state I_2_. The distances between F486 (SARS2) or L472 (SARS) and ACE2 hydrophobic center in different states are shown in **g**. **h**, **i** Lifetimes of force-dependent bonds between ACE2 and SARS2-RBD variants with mutations that abolish force-induced I_1_ (**h**) or I_2_ (**i**) intermediate state (solid plots) in comparison with that of SARS2-RBD^WT^ (purple dashed plots). All error bars represent SEM. 0.01 < *P < 0.05 and 0.0001 < ***P < 0.001 by Student’s *t*-test.

To further test this hypothesis, we then aimed to identify key residues for regulating the stability of two intermediate states and for selecting the dissociation pathways in ACE2 interacting with SARS2-RBD^WT^ or SARS-RBD^WT^. We examined the residues located at RBD/ACE2 binding interface that were essential for forming either I_1_ or I_2_ states. For SARS2-RBD^WT^, force promotes hydrogen bond (H-bond) formation of its Q493 with ACE2-K31 when switching from the I_0_ to I_1_ state (Fig. 3a, b, d; Supplementary information, Fig. S6a); for SARS-RBD^WT^, corresponding residue N479 either forms H-bond or not with ACE2-K31 in the I_0_ state of P_1_ or P_2_ pathway respectively, and force does not change N479 binding state with ACE2-K31 when switching from the I_0_ to I_1_ state in both pathways (Fig. 3c, d; Supplementary information, Fig. S6b, c). For the I_2_ state, it is defined by the interaction between SARS2-RBD^WT^ RBM and ACE2. The interaction, mainly formed by the hydrophobic packing of SARS2-RBD^WT^-F486 with the hydrophobic center formed by L79, M82, and Y83 of ACE2, solely maintains SARS2-RBD^WT^/ACE2 binding after the force-induced RBD rotation (Fig. 3e, g; Supplementary information, Fig. S6d). In contrast, for SARS-RBD^WT^, these interactions are unstable even in the absence of force (Supplementary information, Fig. S6e, f), as they frequently switch between bound (55% of times) and unbound (45% of times) states (Fig. 3f). The bound state favors the P_1_ pathway (75% of occurrence frequency), whereas the unbound state more likely (80% of occurrence frequency) leads to the P_2_ pathway for faster RBD/ACE2 dissociation (Fig. 3f). Simulations on F486L mutant (SARS2-RBD^F486L^) further reveal unstable SARS2-RBD^F486L^/ACE2 associations (Supplementary information, Fig. S7) in the absence of force, confirming the importance of F486 in maintaining RBD/ACE2 mechanical stability. Moreover, both SARS2-RBD Q493N and F486L mutations reduce the force-dependent bond lifetime of SARS2-RBD/ACE2 interaction, shortening the maximum bond lifetime almost by two to three folds (Fig. 3h, i; Supplementary information, Fig. S3e, f). Together, these results collectively suggest a model of mechano-enhanced viral infection in which tensile mechanical force can enhance spike binding with ACE2 to foster viral infection.

### Mechano-enhanced SARS2-S/ACE2 binding fosters viral infection

**Fig. 4:**
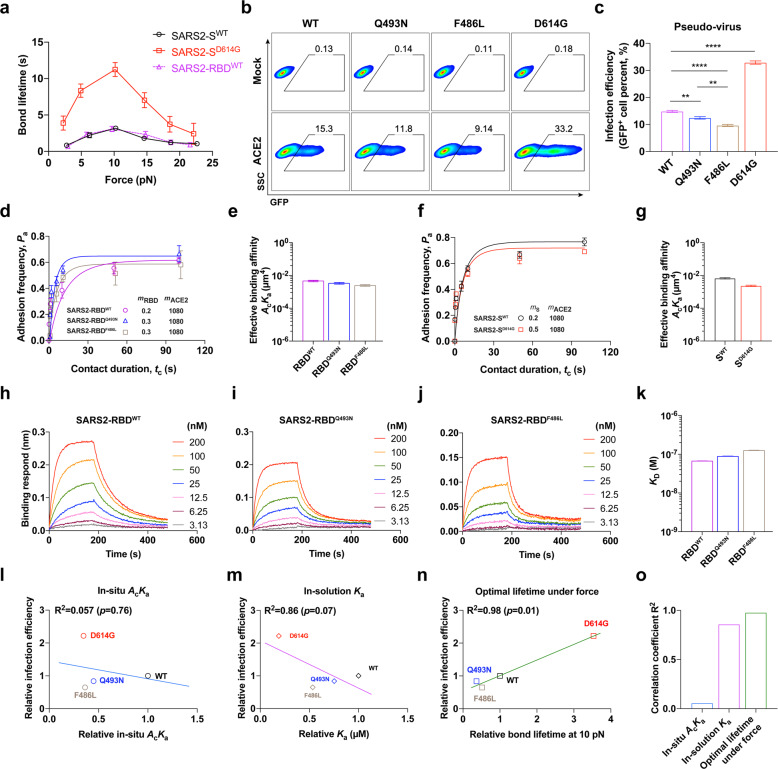
Mechano-enhanced SARS2-S/ACE2 binding fosters viral infection. **a** Force-dependent bond lifetimes of SARS2-S^WT^ (black solid plots) or SARS2-S^D614G^ (red solid plots) binding with ACE2, in comparison with SARS2-RBD^WT^ (purple dashed plots). Error bars represent SEM. **b**, **c** Pseudovirus infection of SARS2 wild-type (WT) or mutants (Q493N, F486L and D614G). Representative flow cytometry analysis of GFP in ACE2-expressing 293T cells infected with SARS2 pseudovirus (**b**). Comparisons of the efficiencies of SARS2 pseudotyped viruses (WT, Q493N, and F486L) infecting ACE2-expressing cells (**c**). All error bars represent SEM. 0.001 < **P < 0.01 and ****P < 0.0001 by Student’s *t*-test. **d**–**g** The affinity determination of SARS2-RBD mutants binding with ACE2 by adhesion frequency assay. Adhesion frequency curves of ACE2 binding with SARS2-RBD mutants (Q493N and F486L) (**d**) and SARS2-S (SARS2-S^WT^ and SARS2-S^D614G^) are shown (**f**). Molecular surface densities (number/μm^2^) of ACE2 and SARS2-RBD or SARS2-S are indicated. The corresponding effective binding affinities (*A*_c_*K*_a_) (**e** and **g**) are plotted and compared, respectively. **h**–**k** The affinity determination of SARS2-RBD mutants binding with ACE2 by biolayer interferometry. The representative set of curves of ACE2 binding with SARS2-RBD mutants (WT (**h**), Q493N (**i**), and F486L (**j**)) are shown. The corresponding equilibrium dissociation constant (*K*_D_) (**k**) is calculated by 1:1 binding model. **l**–**o** The correlation analysis of the infection efficiencies of SARS2 pseudoviruses with SARS-S/ACE2 in-situ affinity (**l**), in-solution affinity (*K*a = 1/*K*_D_) (**m**) and bond lifetime at 10 pN (**n**). Their respective correlations with the corresponding pseudovirus infectivity are compared (**o**). All error bars represent SEM.

To further test this model, we next focused on examining how a spike mutant with higher viral infectivity (SARS2 spike D614G variant, SARS2-S^D614G^) is impacted by mechano-regulation. SARS2 with spike D614G variation was more epidemic with enhanced replication and transmission than that without this variation.^38–41^ D614G mutation was reported to decrease S1/S2 cleavage^42^ and increase incorporation of the spike into the pseudo-virion.^42,43^ However, the authentic virion does not demonstrate these phenotypes,^44,45^ suggesting that the changed level of S1/S2 cleavage or spike incorporation into the virion is not a convincing explanation for the higher infectivity of the D614G mutant. Moreover, SARS2-S^WT^ and SARS2-S^D614G^ have comparable binding affinities to ACE2 as both slightly increased and decreased affinities of the mutant to ACE2 were reported.^39,42,46–48^ Considering that D614G variation makes RBD more flexible and more readily to adopt an up conformation,^39^ we hypothesized that D614G mutation might affect force-dependent regulation of the SARS2-S/ACE2 bond lifetime. Indeed, we found that SARS2-S^D614G^ bound ACE2 much more strongly than SARS2-S^WT^ under force with an almost four-time longer optimal lifetime (11.2 s for SARS2-S^D614G^ vs 3.2 s for SARS2-S^WT^) under the 10 pN optimal force (Fig. 4a; Supplementary information, Fig. S3g–i). In contrast, SARS2-S^WT^, SARS2-S1^WT^, SARS2-S1^D614G^, and SARS2-RBD^WT^ bind ACE2 with almost the same force-dependent bond lifetimes (Fig. 4a; Supplementary information, Fig. S8). These results demonstrate that D614G variation enhances force-dependent SARS2-S recognition of ACE2.

Functionally, the D614G variant exhibits higher pseudovirus (the HIV-based lentivirus pseudotyped with SARS2-S) infectivity to ACE2-expressing cells than WT (Fig. 4b, c), consistent with previous reports.^38–40^ This increased infectivity of the D614G variant can be explained by a longer force-dependent bond lifetime of SARS2-S^D614G^ than WT in binding with ACE2, despite their similar in-solution or in-situ binding affinities to ACE2^39^ (Fig. 4f, g).

Similarly, SARS2-RBD Q493N and F486L mutants, which show similar binding affinities but shorter force-dependent bond lifetimes than WT in the interaction with ACE2 (Figs. 3h, i; 4d, e, h–k; Supplementary information, Fig. S3e, f; Table S1), significantly attenuate the pseudovirus infection (Fig. 4b, c), further suggesting that force-dependent bond lifetime of SARS2-RBD/ACE2 is a better predictor for SARS2’s infectivity.

Taken together, our data demonstrate that the mechano-regulated dissociation kinetics (e.g., optimal lifetime at 10 pN) of SARS2-S with ACE2 best correlates with viral infection efficiency (Fig. 4n, o), in contrast to the in-situ or in-solution binding affinity (Fig. 4l, m), suggesting the essential role of force-strengthened spike/ACE2 binding in regulating SARS2 viral infection.

### Mechanical force dramatically accelerates SARS2-S S1/S2 detachment

**Fig. 5:**
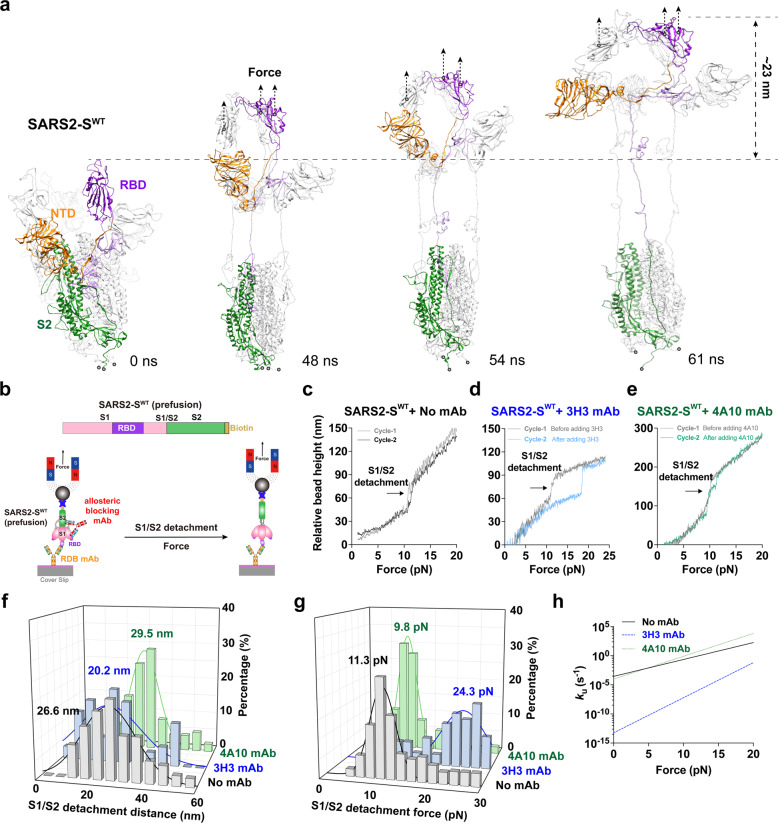
Mechanical force dramatically accelerates SARS2-S S1/S2 detachment, which is significantly impeded by an S1/S2-binding and neutralizing antibody derived from COVID-19 patients. **a** Sequential SMD snapshots of SARS2-S^WT^ S1/S2 detachment under pulling force. The anchoring and force pulling residues (gray ball), force direction (black dashed arrow), and timestamps for all snapshots are indicated. **b** Schematic diagram of the design of SARS2-S^WT^ with a C-terminal biotin tag (up panel) for single-molecule MT pulling experiments (bottom panel). **c**–**h** Representative force stretching curves from single-molecule MT pulling experiment to demonstrate force-induced S1/S2 detachment of SARS2-S^WT^ in the absence (**c**) or presence of neutralizing (3H3) (**d**) or non-neutralizing S2-binding antibody (4A10) (**e**). Their respective histograms of S1/S2 detaching distances (**f**) and forces (**g**) are compared. The mean values (matched colors) obtained by Gaussian fitting are indicated respectively. Their force-dependent detachment rates derived from Bell model are compared in **h**.

As S1/S2 detachment is an essential step preceding SARS2-S S2 structural rearrangement and fusion machinery formation, we further explored whether mechanical force transmitted by RBD/ACE2 interaction could drive and accelerate the detachment process. Using SMD simulations, we pulled a spike trimer on its RBDs (Fig. 5a; Supplementary information, Video S5) and observed that mechanical force indeed decreased the contact area between S1 and S2 from ~450 nm^2^ to ~0 nm^2^, leading to rapid S1/S2 detachment (Supplementary information, Fig. S9a). Consequently, SARS2-S^WT^ extended ~23 nm in the direction of force application (Supplementary information, Fig. S9b), which was further validated and confirmed with our single-molecule magnetic tweezers (MT) pulling experiments (Fig. 5b, c). Single SARS2-S^WT^ presents a pronounced conformational extension (~26.6 nm) mostly under ~11.3 pN pulling force (Fig. 5f, g). Based on Bell model,^49^ the S1/S2 detachment rate (or unfolding rate, *k*_u_) of SARS2-S^WT^ is 2.9 × 10^–4^ s^−1^ in the absence of force, suggesting that S1/S2 detachment is unlikely to occur spontaneously. Instead, we found that only 10 pN tensile force could drastically increase the detachment rate to 0.2 s^−1^ (Fig. 5h), almost 1000 times faster than that in no-force condition, further demonstrating the essential role of mechanical force on activating and accelerating S1/S2 rapid detachment.

### An S1/S2-binding non-RBD-blocking antibody significantly impedes mechano-accelerated SARS2-S S1/S2 detachment

Unexpectedly, we identified a non-RBD-blocking monoclonal antibody (mAb, clone 3H3, derived from convalescent COVID-19 patients), which was reported to bind both S1 and S2 subdomains of the spike protein and have high neutralization activity against SARS2 infection through ACE2 with no clear mechanisms.^50^ Single-molecule MT pulling experiments showed that this antibody significantly impedes S1/S2 detachment. Moreover, in the presence of 3H3 mAb, a larger force (~24.3 pN) on average was required to induce a shorter extension distance (~20.2 nm) needed for SARS2-S^WT^ S1/S2 detachment (Fig. 5d, f), dramatically decreasing S1/S2 detachment rate by ~3 × 10^6^ folds from 0.2 s^−1^ to 6.2 × 10^–8^ s^−1^ under ~10 pN force (Fig. 3h). This clearly suggests that 3H3 mAb locks S1/S2 subunits together to stabilize SARS2-S even under force loading, potentially preventing follow-up fusion machinery formation and viral invasion. In contrast, another non-RBD-blocking, S1/S2-binding, and non-neutralizing mAb (clone 4A10, also derived from convalescent COVID-19 patient),^50^ hardly affects S1/S2 detachment (Fig. 5e–h). Collectively, our SMD simulation analysis and single-molecule measurements demonstrate that mechanical force dramatically accelerates S1/S2 detachment, which can be prevented by a neutralizing mAb targeting S1/S2.

### D614G variation accelerates force-induced S1/S2 detachment

**Fig. 6:**
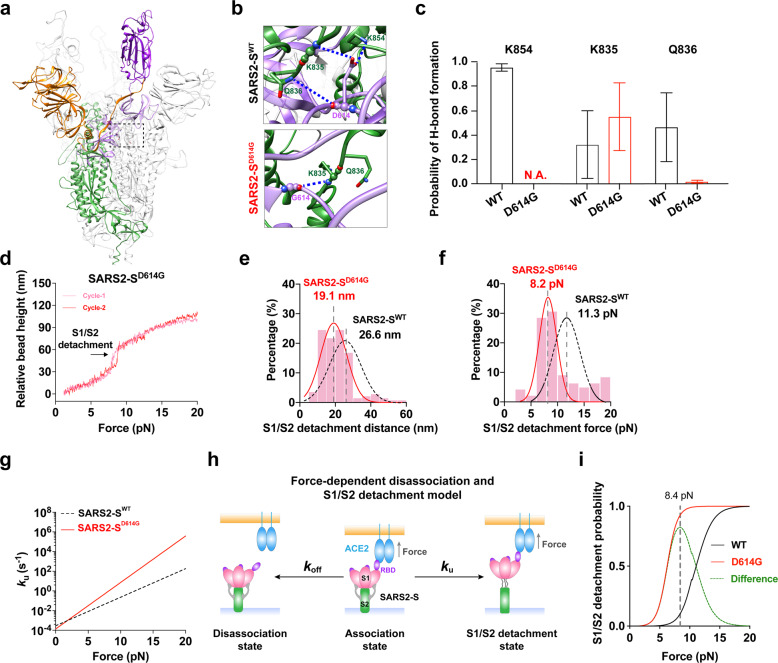
D614G variation further accelerates S1/S2 detachment under mechanical force. **a**–**c** H-bond network analysis of G614 interacting with residues in its vicinity (marked with a black dashed box in **a** and zoomed-in views in **b**) in the structure of SARS2-S^D614G^. The probabilities of H-bond formation in WT and D614G mutant are compared (**c**). All error bars represent SEM. **d** Representative force stretching curves from single-molecule MT demonstrating force-induced S1/S2 detachment of SARS2-S^D614G^. **e**–**g** S1/S2 detaching distance (**e**), force (**f**), and detachment rate (**g**) of SARS2-S^D614G^ (red solid plots) are respectively compared with those of SARS2-S^WT^ (black dashed plots). **h** Schematic diagram of the force-dependent SARS2-S activation model. **i** Comparison of S1/S2 detaching probability of SARS2-S^WT^ with that of SARS2-S^D614G^ under force.

For SARS2-S^WT^, residue D614 forms hydrogen bonds with residues on the S2 subunit of neighboring protomer to keep S1/S2 tight assembly. The D614G variation reduces the number of interdomain hydrogen bonds (Fig. 6a, b), specifically abolishing two hydrogen bonds between D614 and K854 or Q836 (Fig. 6b, c). This led us to hypothesize that D614G variation might weaken S1/S2 association. We next performed single-molecule pulling experiments with MT to characterize S1/S2 mechanical stability in the presence of the D614G variation. Interestingly, a shorter S1/S2 detaching distance on average (~19.1 nm) was observed for a single SARS2-S^D614G^ (Fig. 6d, e), suggesting that the D614G variation partially impairs S1/S2 assembly. Compared with 11.3 pN force to detach S1/S2 in SARS2-S^WT^, a much smaller tensile force (~8.2 pN) is required to detach S1 and S2 in SARS2-S^D614G^ (Fig. 6d, f), drastically increasing the detachment rate by 35 times from 0.2 s^−1^ (for SARS2-S^WT^) to 7.2 s^−1^ under ~10 pN force (Fig. 6g). These results suggest that S1/S2 subunits in SARS2-S^D614G^ are less mechanically stable than those in SARS2-S^WT^. Integrating force-dependent spike/ACE2 disassociation and S1/S2 detachment kinetics, we built up a kinetic model and revealed that the D614G variant with stronger force-dependent ACE2 binding not only accelerated S1/S2 detachment but also had an 8-time higher probability than WT to make this detachment occur (0.92 for D614G vs 0.1 for WT at 8.4 pN) (Fig. 6h, i), providing an unprecedented quantitative kinetic evidence and molecular mechanism to explain higher infectivity of the D614G variant (Fig. 4b, c).

## Discussion

Utilizing single-molecule biophysical approaches, molecular dynamics (MD) simulation, and pseudovirus infection assay, we demonstrate mechanical activation of SARS2-S upon binding to ACE2 and the subsequent S1/S2 detachment for priming S2 fusion machinery. Our findings indicate that SARS2 exploits the mechanical cues to enhance their invasion into host cells by mechanically strengthening its spike binding with host ACE2 receptors and by accelerating S1/S2 detachment to destabilize the pre-fusion spike trimer. Our findings suggest that mechano-activation of SARS2-S is essential to trigger structural rearrangement of SARS2-S and to promote its transition to the post-fusion state to facilitate successful viral fusion. Impairment of inter-protomer interactions by the D614G variation not only strengthens force-dependent SARS2-S/ACE2 binding, but more importantly, induces S1/S2 detachment much faster than in the WT protein under force, providing a new molecular explanation for the high infectivity of the SARS2 D614G variant. A non-RBD-blocking but S1/S2-binding neutralizing mAb derived from convalescent COVID-19 patients dramatically impedes S1/S2 forced-detachment, revealing an unprecedented virus-neutralizing strategy for therapeutic antibody development.

It is the first time to demonstrate that mechanical force prolongs bond lifetime of viral spike binding with host receptors. Our finding of force-strengthened spike/ACE2 binding provides molecular evidence to support the notion that the attachment of SARS2 virion on the host-cell PM survives longer than SARS in a biomechanical environment. This is reminiscent of the previous observations that mechanical force favors membrane fusion and endocytosis,^22,51,52^ which are two common routes utilized by the coronavirus to enter host cells.^10,11,53,54^

As receptor binding and the follow-up S1/S2 detachment are both essential to trigger the S2 structural rearrangement and fusion machinery formation for the effective viral infection,^10–12^ SARS2-S-binding mAbs with neutralization potency are applied for therapeutic interventions of COVID-19. While most of the studies focused on searching for neutralizing mAbs that block SARS2-S-RBD/ACE2 binding,^55–57^ our findings identify an alternative neutralizing strategy that exploits non-RBD-blocking but S1/S2-locking antibodies to stabilize SARS2-S structure by preventing S1/S2 detachment and follow-up S2 fusion machinery formation. Such strategy potentially can compensate or complement receptor-blocking strategy no matter what other novel spike receptor is found.^6,58^ This finding also suggests that mAbs targeting S1/S2 epitopes and restraining S1/S2 detachment may provide high neutralization potency against SARS2 infection by inhibiting pre-fusion-to-post-fusion transition of SARS2-S.

Interestingly, SARS2-S^D614G^ exhibits much stronger force-dependent binding than SARS2-S^WT^ with ACE2. As the D614G variation disrupts stable contact between inter-protomers to allosterically favor more SARS2-RBD up conformation,^39^ this enhancement of force-dependent recognition might be due to the synergistic effect of two or three up RBDs in a single SARS2-S trimer. Although the detailed molecular mechanism for such a synergetic effect is still unclear, there are several possible explanations. One plausible explanation is that the S1 subunits of all three protomers in the D614G variant are more flexible such that it may release the spatial restriction to allow more than one RBD binding with ACE2 dimer simultaneously. Another alternative explanation is that ACE2 sequentially binds with each up RBD of SARS2-S^D614G^ via sliding-rebinding mechanism.^59^ Also, we can hardly rule out the possibility that D614G variation may cause a larger extent of force-induced rotational conformational changes of RBD, thereby resulting in a longer force-dependent bond lifetime of SARS2-S/ACE2 binding.

**Fig. 7:**
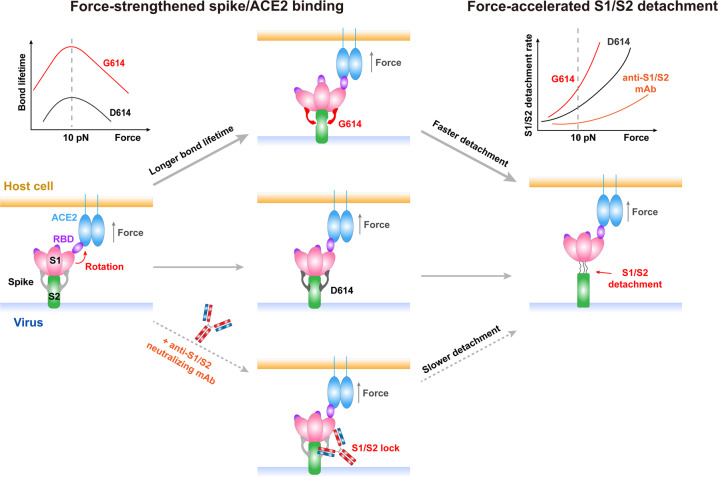
A model for mechano-activation of SARS2-S and its inhibition by a non-RBD-blocking neutralizing antibody targeting S1/S2. Mechanical force strengthens SARS2 spike binding with host ACE2 receptors and accelerates its S1/S2 detachment to facilitate viral invasion. Impairment of inter-protomer interactions by the D614G variation not only strengthens force-dependent SARS2-S/ACE2 binding, but also accelerates force-induced S1/S2 detachment. S1/S2-locking antibodies stabilize SARS2-S structure and dramatically impede S1/S2 force-induced detachment, neutralizing SARS2.

To conclude, we demonstrate that mechanical force counter-intuitively impedes SARS2-S/ACE2 dissociation and induces subsequent S1/S2 rapid detachment for effective viral infection, and that D614G variation further enhances this mechano-regulation to increase SARS2 infectivity. Our results also reveal an unexpected virus-neutralizing mechanism of a non-RBD-blocking antibody from COVID-19 patients via preventing force-regulated S1/S2 detachment (Fig. 7). Thus, our findings not only answer key questions on whether and how mechanical cues impact SARS2 viral entry and infection, but also provide valuable insights into the force-dependent dynamic spike/ACE2 interaction and the follow-up S1/S2 detachment. All of these would shed lights on the development of better therapeutics targeting the mechano-sensitive motifs for COVID-19 treatment.

We would like to point out that our present study contains a few potential limitations. First, all single-molecule assays performed here were with a widely used soluble recombinant SARS2-S that differs from the native protein in three aspects: ‘GSAS’ and ‘PP’ substitutions at residues 682–685 and 986–987, and synthetic trimerization helix substitution at the transmembrane domain, were introduced to stabilize the trimer. Thus, the structures of the native spike might differ from what we observe in the context of the ectodomain. Second, we propose that the D614G variation of SARS2-S could allosterically strengthen its binding with ACE2 under force and simultaneously enhance the force-accelerated S1/S2 detachment, which might favor effective viral infection. However, our experiments do not rule out other possibilities as potential mechanisms.^42–45^ Third, confirmative experimental evidence that mechanical force directly affects SARS2 infection in a setting of authentic SARS2 and live cells is still absent, although we demonstrate that SARS2 exploits mechanical force to impede spike/ACE2 dissociation and accelerate subsequent S1/S2 detachment for effective pseudovirus infection. Fourth, all the SARS2-S mutant infection experiments were performed using pseudovirus infection models in established cell lines, and therefore the results obtained need to be confirmed by authentic SARS2 virus infection experiments in the future. Finally, we propose an alternative neutralizing strategy, the S1/S2-locking neutralization antibody, which also needs to be further investigated.

## Materials and methods

### Plasmid construction

The plasmids for recombinant protein purification were constructed by inserting the cDNA sequences of SARS2-RBD (residues: 333–527), SARS-RBD (residues: 320–513), SARS2-S1 (residues: 1–685) and SARS2-S (residues:1–1208) into the pHAGE vector using the ClonExpress Ultra One Step Cloning Kit (Cat. C115, Vazyme, China). SARS2-RBD and SARS-RBD recombinant protein plasmids each contains an N-terminal Igκ leader signal peptide plus Flag tag and a C-terminal AviTag plus 6× His tag. SARS2-S recombinant protein plasmid contains ‘GSAS’ and ‘PP’ substitutions at residues 682–685 and 986–987, a C-terminal T4 fibritin trimerization motif and AviTag plus 6× His tag. F486L, Q493N and D614G mutations were introduced by PCR-mediated mutagenesis by Phanta Master Mix (Cat. #P511, Vazyme, China). The plasmid for cell line construction was constructed by cloning full-length ACE2 cDNA sequence (inserting HA tag after signal peptide) into the pHAGE vector.

### Protein expression and purification

Expi293F cells (Cat. #A14527, Thermo Fisher) were used for recombinant protein expression. pHAGE plasmids containing recombinant protein coding sequences were transiently transfected into the cultured cells by polyethylenimines (PEI) (Cat. #23966, Polysciences). After 5 days of expression, the supernatants were collected, centrifuged, and concentrated through VivaFlow 200 flipflow filtration MWCO 30 kDa (Sartorius). Soluble recombinant proteins in the concentrated mixture were purified through HisTrap HP (GE Healthcare) and HiTrap Q HP column (GE Healthcare) affinity chromatography column. Then AviTag peptide of the recombinant protein was covalently labeled with biotin through BirA enzymatic biotinylation reaction. Finally, the recombinant protein was further purified via Superdex 75 10/300 GL or Superdex 200 Increase (GE Healthcare) gel filtration chromatography with phosphate buffer saline (pH = 7.4) composed of 2 mM KH_2_PO_4_, 8 mM Na_2_HPO_4_, 136 mM NaCl, 2.6 mM KCl.

### Cell line construction

ACE2 was expressed in U937 cells by lentivirus infection. The lentivirus was produced through co-transfection of pHAGE plasmid, psPAX2 and pMD2.G into HEK 293T cells. The U937 cells with similar expression levels of ACE2 were selectively sorted and collected through flow cytometry sorting (Beckman).

### Recombinant protein-coated microspheres/RBC preparation

The recombinant protein-coated microspheres and red blood cells (RBCs) were prepared according to previously published methods.^60–64^ Briefly, for force-clamp assay, borosilicate glass microspheres (Cat. #9002, Thermo Fisher) were first chemically modified with -SH group through 3-mercapto-propyl-trimethoxysilane (Cat. #175617, Sigma-Aldrich), then incubated with streptavidin-maleimide (Cat. #S9415, Sigma-Aldrich) overnight in 200 mM phosphate buffer (pH = 6.7) at room temperature (RT). For the adhesion frequency assay, human RBCs were directly reacted with biotin-PEG3500-NHS (Cat. #62717, JenKem, China) at RT for 30 min in 10 mM HEPES buffer (containing with 145 mM NaCl, roughly 300 mOsm osmotic pressure, pH = 7.4) and then coated by streptavidin in HEPES buffer containing 1% BSA at RT for 30 min. Finally, suitable biotin-labeled recombinant protein was coated on microspheres or RBCs through biotin-streptavidin reaction in HEPES buffer containing 1% BSA at RT for 30 min.

### Force-clamp assay

The detailed experimental procedure was previously described.^61,62,64^ In brief, human RBCs attached with recombinant protein-coated microsphere, as a pN-level force sensor, were held by a micropipette on the left. An ACE2-expressing U937 cell was aspirated by the other micropipette, whose movement was controlled through a linear piezoelectric actuator (Physic Instrument) with sub-nanometer precision. For the bond lifetime measurement, an ACE2-expressing U937 cell was driven to approach to and contact recombinant protein-coated microsphere. After 0.1 s of contact, the cell was retracted at 1000 pN/s and clamped at a preset force until the bond broke. The bond lifetime was defined as the duration of the clamped phase. To ensure that ~90% bond lifetimes were generated from a single bond, the adhesion frequency was kept < 20% by adjusting recombinant protein densities on the microspheres. All above experiments were conducted in a chamber filled with ~500 μL DMEM medium containing 0.5% BSA.

### Molecular stiffness determination

The molecular stiffness was measured by the single-molecule force spectroscopy on BFP, which has been described in details previously.^65,66^ In brief, the stiffness of the spike/ACE2 bond (*k*_m_) could be calculated from the slope of the force versus displacement curve, which was obtained during the retraction step of each BFP event (Supplementary information, Figs. S2a, S3a). When F < 0 pN, the slope of the curve represents the stiffness of the cell (*k*_c_, assuming that spike/ACE2 is incompressible). When F > 0 pN, the slope of the curve represents the stiffness (*k*_s_) of the serially connected system, containing a single spike/ACE2 bond and the cell. According to Hooke’s law, we can calculate *k*_m_ = 1/(1/*k*_s_ − 1/*k*_c_) for each force vs displacement curve.

### Adhesion frequency assay

The force-free in-situ binding kinetics between recombinant proteins and ACE2 was measured by adhesion frequency assay, as previously described.^60,63^ An ACE2-expressing U937 cell was driven through a linear piezoelectric actuator to contact recombinant protein-coated RBC for a preset contact duration (*t*_c _= 0.1, 0.5, 1, 5, 10, 50 and 100 s), and retracted to judge adhesion event occurrence by RBC membrane deformation. After 50 contact–retract cycles, adhesion frequency (*P*_a_) was calculated. Then the obtained *P*_a_ and *t*_c_ curve was fitted with the following kinetic equation:

$$P_{{{{{{{\mathrm{a}}}}}}}} = 1 - exp\left\{ { - m_{{{{{{{{\mathrm{ACE}}}}}}}}2}m_{{{{{{{{\mathrm{spike}}}}}}}}}A_{{{{{{{\mathrm{c}}}}}}}}K_{{{{{{{\mathrm{a}}}}}}}}\left( {1 - exp\left( {k_{{{{{{{{\mathrm{off}}}}}}}}}t_{{{{{{{\mathrm{c}}}}}}}}} \right)} \right)} \right\}$$

*m*_ACE2_ and *m*_spike_ represent the molecular density of ACE2 and SARS2-RBD, SARS-RBD or SARS2-S recombinant protein respectively, which were calculated through flow cytometry and Quantum™ MESF beads (Bangs Laboratories, Inc.). *A*_c_*K*_a_ and *k*_off_ were denoted as effective in-situ affinity and in-situ off-rate in a force-free condition, respectively. The effective in-situ on-rate *A*_c_*k*_on_ was calculated using the following kinetic equation:

$$A_{{{{{{{\mathrm{c}}}}}}}}k_{{{{{{{{\mathrm{on}}}}}}}}} = A_{{{{{{{\mathrm{c}}}}}}}}K_{{{{{{{\mathrm{a}}}}}}}} \times k_{{{{{{{{\mathrm{off}}}}}}}}}$$

### Biolayer interferometry (BLI) binding assay

BLI binding assay was performed through Octet RE96E instrument (ForteBio), which was supported by Sky-bio Co., Ltd. in Hangzhou. In brief, 25 μg/mL Fc-tagged ACE2 recombinant protein (Cat. #ACE-HM501, Kactus Biosystems Co., Ltd., China) was loaded onto Protein A (ProA) Biosensors (Cat. #18–5010, ForteBio) for 1500 s. Free ACE2 was washed out by a 180-s wash with kinetics buffer (PBS, 0.05% Tween-20, 0.1% BSA, pH = 7.4). Then the SARS2-RBD recombinant protein with different concentrations was loaded to associate with immobilized ACE2 for another 180 s. Finally, kinetics buffer was used to dissociate SARS2-RBD from ProA Biosensors for 300 s. The corresponding binding affinity (*K*_D_) was calculated by a 1:1 binding model.

### Magnetic tweezers setup and chamber preparation

The details about the home-made MT setup, force calibration and experimental design were recently published.^21^ Briefly, a piece of coverslip (Cat. #12–545-B, Thermo Fisher) was sequentially cleaned with sonication in Decon90 detergent, acetone, isopropanol and deionized water. The coverslip was then thoroughly dried in a 120 °C oven and further cleaned in O_2_ plasma cleaner for 5 min. Next, the coverslip surface was modified with NH_2_ group by 1% (3-aminopropyl) triethoxysilane (APTES, Cat. #A107147, Aladdin, China) in methanol for 1 h. The coverslip was then sequentially washed twice with methanol and deionized water before thoroughly dried in a 120 °C oven. The coverslip was packed with another clean coverslip without NH_2_-modification in a hamburger pattern with two strips of parafilm to form the experimental chamber.

### Single-molecule pulling of SARS2-S with MT

The single-molecule pulling experiment of SARS2-S was performed to probe S1/S2 detachment through MT. First, the chamber was functionalized with CHO group by 0.5% glutaraldehyde solution for 1 h and then washed twice by PBS buffer. The functionalized chamber was then incubated with 50 μg/mL SARS2-RBD mAb (Cat. #AHA001, Sanyou Biopharmaceuticals, China) for 15 min, and 50 μL polystyrene bead (Cat. #17145-5, polysciences) solution (5 × 10^7^/mL) was added into the chamber for incubation overnight. The potential non-specific interaction in the chamber was then blocked by 2% BSA for 4 h. Approximately 1 pg/mL SARS2-S in 1% BSA solution was vortically incubated with streptavidin-coated magnetic beads (Cat. #65305, Thermo Fisher) for 30 min. Finally, SARS2-S-coated magnetic beads were injected into the chamber and kept for 20 min to allow the beads to be captured by the SARS2-RBD mAb on coverslip surface before single-molecule MT pulling experiments.

For single-molecule pulling experiments, the successfully linked tether (SARS2-S^WT^ or SARS2-S^D614G^) was pulled from 0 to 30 pN with a constant force loading rate of 1 pN/s, then released to 10 pN with –5 pN/s and finally to 0 pN with –0.5 pN/s. These sequential steps together are defined as a force cycle. Between two adjacent force cycles, there was a 60-s waiting time for SARS2-S to thoroughly refold. The S1/S2 detachment forces and distances were collected from hundreds of pulling events of dozens of independent tethers.

For single-molecule pulling of SARS2-S^WT^ in the presence of neutralizing mAb, after a successfully linked tether was determined by testing the S1/S2 detachment force and distance, 270 nM neutralizing mAb (clone: 3H3 or 4A10) was gently and very slowly injected into the experimental chamber for 15-min incubation and the same tether continued to be measured for the S1/S2 detachment force and distance in the presence of neutralizing mAb. All the above single-molecule pulling experiments were performed in PBS with 1% BSA.

### Force-dependent S1/S2 detachment rate calculation

The force-dependent S1/S2 detachment rate of SARS2-S could be described and calculated based on Bell’s model.^49^ Briefly, the S1/S2 detachment rate of SARS2-S was calculated by fitting detachment force histogram to the following equation:^67^

$$P^{(F)} = \frac{{k_0}}{r}exp\left\{ {\frac{{{\Delta} xF}}{{k_{{{{{{{\mathrm{B}}}}}}}}T}} + \frac{{k_{{{{{{{\mathrm{B}}}}}}}}Tk_0}}{{{\Delta} xr}}\left[ {1 - exp\left( {\frac{{{\Delta} xF}}{{k_{{{{{{{\mathrm{B}}}}}}}}T}}} \right)} \right]} \right\}$$

Where *P*^*(F)*^ is the probability of detachment force from histogram, *k*_0_ is the detachment rate at zero force, *r* is the force loading rate, *Δx* is the transition distance of spike between original and transition states, *F* is the detachment force, *k*_B_ is Boltzmann’s constant, *T* is the absolute temperature, *k*_B_*T* is approximately 4.1 pN·nm. With all above known data, *Δx* and *k*_*0*_ were calculated by fitting with the above equation.

Once *Δx* and *k*_*0*_ were obtained, the S1/S2 detachment rate of SRAS2-S at any force can be predicted by the following equation:^49,67^

$$k_u = k_0exp\left( {\frac{{{\Delta} xF}}{{k_{{{{{{{\mathrm{B}}}}}}}}T}}} \right)$$

Where *k*_u_ is the force-dependent detachment rate.

### Pseudovirus preparation and infection

SARS2-GFP pseudo-viruses were generated by co-transfecting envelop plasmid (pCAG-SARS2-S∆C19), package plasmid (PLP1 and PLP2) and transfer plasmid (pCDH-CMV-CopGFP) into HEK 293 T cells using PEI, and were harvested at 50 h post-transfection. For pseudovirus infection, 2 × 10^5^ cells were seeded into a 24-well plate. After 12 h culture, the crude virus was used to infect the ACE2-expressing HEK 293 T cells. The culture medium was changed 12 h later and cells were incubated for an additional 36 h before analysis by FACS to check GFP expression level.

### MD simulations and SMD simulations on RBD/ACE2 complex

The crystal structures of ACE2-PD (ACE2) in complex with SARS2-RBD^WT^ (PDB codes: 6LZG,^25^ 6M0J^32^) or SARS-RBD^WT^ (PDB codes: 2AJF,^68^ 3SCI^69^) were used as the starting models in MD simulations. The complex models of ACE2 and SARS2-RBD^F486L^ were generated based on SARS2-RBD^WT^/ACE2 structures with the MUTATE plugin in VMD. These initial models were rotated to make their long axis (the line linking C-terminal of ACE2 and C-terminal of RBD) parallel to the *x*-axis, and then processed with VMD PSFGEN plugin to add hydrogen atoms and other missing atoms. The resulted systems were solvated in rectangular water boxes with TIP3P water model. Na^+^ and Cl^–^ ions were then added to these solvated systems to neutralize the systems and maintain salt concentration at ~150 mM.

All systems were first equilibrated with four steps: (1) 10,000 steps energy minimization with the heavy atoms of proteins fixed, followed by 2-ns equilibration simulations under 1-fs timestep with these atoms constrained by 5.0 kcal/mol/Å^2^ spring; (2) 10,000 steps energy minimization with the heavy atoms of proteins fixed, followed by 2-ns equilibration simulations under 1-fs timestep with these atoms constrained by 1.0 kcal/mol/Å^2^ spring; (3) 2-ns equilibration simulation under 1-fs timestep with the heavy atoms of proteins constrained by 0.2 kcal/mol/Å^2^ spring; (4) 10-ns equilibration simulation under 1-fs timestep without constrains. Subsequently, ~400-ns production simulations were carried out with 2-fs time steps under rigid bond algorithms, and the snapshots were saved every 40 ps for further analysis. During the simulations, the temperature of each system was maintained at 310 K with Langevin dynamics and the pressure was controlled at 1 atm with the Nosé-Hoover Langevin piston method.^70^ Particle Ewald Mesh summation was used for electrostatic calculation and a 12 Å cutoff with 10 to 12 Å smooth switching was used for short-range non-bounded interactions.

Representative snapshots of the production runs of each system were chosen, and extra water molecules were appended to extend the box dimension along with *x*-direction to enable complex extension in force-loaded SMD simulations. Before applying forces, these models were first equilibrated with the similar strategy as described above. The final configurations were used for the force-loaded SMD simulations. In each SMD simulation, the C-terminal Cα atom of ACE2 was constrained at its initial position with a dummy spring (spring constant is 2.0 kcal/mol/Å^2^, ~1400 pN/nm) and the C-terminal Cα atom of RBD was pulled with another dummy spring (spring constant is 0.1 kcal/mol/Å^2^, ~70 pN/nm) which moves at ~0.1 nm/ns velocity. The SMD simulations were performed with 1-fs timestep without Langevin temperature and pressure coupling and lasted till the ACE2 and RBD molecules were completely separated, and the snapshots were saved every 20 ps. For SARS2-RBD^WT^ and SARS-RBD^WT^ systems, 18 SMD trajectories were generated in total for the statistical analyses, 9 simulations for each system.

The inter-domain angle (*α*) was used to describe the relative orientation of RBD and ACE2, which was defined as the angle among three centers of mass of heavy atoms of protein: RBD, ACE2/RBD interface (E23–Q42 in ACE2, L492–Q498 in SARS2-RBD^WT^ and W478–I484 in SARS-RBD^WT^) and ACE2. The contact areas between RBD and ACE2, between RBM (residues Q474–C488 for SARS2-RBD^WT^ and F460–C474 for SARS-RBD^WT^) of RBD and ACE2, and between RBD except RBM and ACE2 were calculated. The H-bond networks between ACE2 and RBD were analyzed, the distance threshold of H-bond was set to 3.5 Å between the donor and acceptor atoms, and the angle cutoff was set to 50°. All simulations were performed with NAMD2^71^ software using CHARMM36m force field with the CMAP correction.^72^ The system preparations and trajectory analyses were conducted with VMD.^73^ Illustrations of the representative frames shown in the Figures and the Supplementary Figures were rendered by UCSF Chimera.^74^

### MD simulations on SARS2-S S1/S2 detachment

The crystal structures of SARS2-S^WT^ (PDB codes: 6XR8^27^ and 6VYB^26^) were used as the starting models in MD simulations on force-driven S1/S2 detachment. SARS2-S^WT^ model with full-open conformations (three up RBDs) was generated by reassigned down RBDs to up conformation. The missing regions in structures were modeled by using the homology of SARS-S structures (PDB codes: 6ACC^15^ and 5XLR^75^) or modeled by ModLoop webserver.^76^ The SARS2-S^D614G^ models were generated with the MUTATE plugin in VMD, which was also based on SARS2-S^WT^ (PDB codes: 6XR8^27^ and 6VYB.^26^) After processed with VMD PSFGEN plugin to add hydrogen atoms and other missing atoms, the resulted systems were solvated in the rectangular water boxes with TIP3P water model. Na^+^ and Cl^–^ ions were then added to these solvated systems to neutralize the systems (~150 mM).

All systems were first equilibrated with the similar strategy as described above. Besides, two extra steps were appended before atom constraints were removed, in which 2-ns equilibration simulations were performed under 1-fs timestep with the heavy atoms of protein except for the sidechain atoms of added peptide regions constrained by 0.2 kcal/mol/Å^2^ spring, and followed by 2-ns equilibration with heavy atoms of protein except all atoms of added missing regions constrained by 0.2 kcal/mol/Å^2^ spring. Subsequently, more than 100-ns production simulations were carried out with 2-fs time steps under rigid bond algorithms, and the snapshots were saved every 40 ps for further analyses. Representative snapshots of the production runs of each system were chosen and treated with the similar strategy as described above for SMD simulations of force-driven S1/S2 detachment. In each SMD simulation, Cα atoms of the M900 and A1078 were constrained at their initial positions with a dummy spring (spring constant 2.0 kcal/mol/Å^2^, ~1400 pN/nm) and the V512 Cα atoms of RBD were pulled with another dummy spring (spring constant ~70 pN/nm) which moved at ~0.5 nm/ns velocity.

The contact area between S1 (N-terminal–S680) and S2 (S686–C-terminal) was calculated to demarcate their interaction in the presence and absence of force. The extension of the spike was defined as the distance between constrained atoms and pulling atoms, and the S1/S2 detaching distance, which was set to zero in crystal structure, was used to represent the length changes of spike during the simulations. The number of H-bonds was averaged on three monomers in the spike trimer. During the simulations and trajectories analysis, the key simulation parameters, force field and software were used the same as that in RBD/ACE2 simulations.

### Theoretical estimation of the applying force on the spike/ACE2 bond

Once a virion attaches to the host-cell PM through spike/ACE2 interaction, the interaction potential energy of the virion/host-cell system is reduced. Along with the gradual growth of virion/host-cell contact zone, more spike/ACE2 bonds form, accompanied by a reduction in the interaction potential energy. The reduced interaction potential energy transfers to the bending energy in the bent host-cell PM and the elastic energy in the deformed spike/ACE2 complexes,^23,33,34^ elevating the bending energy of host-cell PM and the elastic energy of deformed spike/ACE2 bonds.

To estimate the forces exerted on the spike/ACE2 bonds, we first considered the force equilibrium of the virion and then the force equilibrium of the virion and host-cell PM system. As the virion bears forces from the spike/ACE2 bonds, the force equilibrium of the virion requires the resultant force from the spike/ACE2 bonds to be zero. For the force equilibrium of virion and host-cell PM system, the total energy includes the energy stored in the bent host-cell PM and that stored in the deformed spike/ACE2 bonds. After the spike/ACE2 bond forms, the host-cell PM bending and the spike/ACE2 bond deformation must satisfy the compatibility condition. According to the theorem of minimum potential energy, the host-cell PM bending and spike/ACE2 bond deformation should reach the lowest total elastic potential energy.

Different types of virus have stiffness ranging from 0.04 to 1 GPa,^35^ while different cells have PM stiffness ranging from 0.1 to a few tens of kPa.^36^ As a result of the significant difference in these stiffnesses, the virus shape change is negligible, and the host-cell PM adopts the shape of the virion during the virus entry. Since the virion contacts the host-cell PM in a rotationally symmetric manner, we used a spherical coordinate to describe the space with the origin at the center of the virion (O in Fig. 1a). Any location on the virion shell can be specified by the polar angle (*φ*) which is defined as an angle between the *z*-axis (the nadir direction) and the vector from the origin (O) to this location. The angle between the *z*-axis and the vector from the O’ to the same location is denoted by *φ*$\prime$. The contact zone can be quantified by the polar angle (*φ*_C_) which is defined as the angle between the *z*-axis and the vector from the origin (O) to the very end of the contact edge (Fig. 1a). For simplicity, we assumed the bent host-cell PM roughly lies on a sphere with the center at O’ and the radius of *R*. The spike/ACE2 bond supports the virion attached to the host-cell PM, leading to a gap between the virion envelope and the host-cell PM. At a certain location *φ* in the contact zone, the gap is equal to the spike/ACE2 bond length (*l*_φ_). The gap at the apex of the virion is denoted by *l*_apex_, which is the same as the length of spike/ACE2 bond at the apex. The radius *R* and gap *l*_apex_ could change when the contact zone grows. With *R* and *l*_apex_, the length of spike/ACE2 bond at *φ* can be described as $l_\varphi = R - r\frac{{\sin \varphi }}{{\sin \varphi^{\prime} }}$ (Fig. 1a), where *r* (45 nm)^30^ is the radius of the virion and:

$$\left\{{\begin{array}{c}{\sin \varphi^{\prime} = \frac{{r \cdot \sin \varphi }}{{\sqrt {\left( {r \cdot \sin \varphi } \right)^2 + \left( {R - r - l_{{{{{{{{\mathrm{apex}}}}}}}}} + r \cdot \cos \varphi } \right)^2} }}} \\ {\cos \varphi^{\prime} = \frac{{R - r - l_{{{{{{{{\mathrm{apex}}}}}}}}} + r \cdot \cos \varphi }}{{\sqrt {\left( {r \cdot \sin \varphi } \right)^2 + \left( {R - r - l_{{{{{{{{\mathrm{apex}}}}}}}}} + r \cdot \cos \varphi } \right)^2} }}} \end{array}} \right.$$

As the virion contacts the host-cell PM in a rotationally symmetric manner, the force on virion is naturally balanced in the *xy*-plane. Thus, we just needed to consider the equilibrium in the *z*-direction. The virion only bears forces through the binding of spike with ACE2. The force magnitude is determined by the deformation of the bond, $$\begin{array}{l}f_\varphi = k_{{{{{{{{\mathrm{mol}}}}}}}}} \cdot {\Delta} l = k_{{{{{{{{\mathrm{mol}}}}}}}}} \cdot \left( {l_\varphi - l_0} \right)\\ \end{array}$$, where *k*_mol_ is the stiffness of the spike/ACE2 bond (~2 pN/nm, see the Molecular stiffness determination section in Materials and Methods; Supplementary information, Fig. S2) and *l*_0_ (23 nm)^77^ is the spike/ACE2 bond length at relaxation, and the direction is along with the bond (Fig. 1a). The component in *z*-direction is the projection of the force $f_{\varphi {{{{{{{\mathrm{Z}}}}}}}}} = f_\varphi \cdot \cos \varphi^{\prime}$. It was previously reported that the average distance between spike molecules is ~15 nm.^30^ One can estimate the density of spike (*n*) on the virion envelope is around ~0.0014 nm^−2^. Thus, the resultant force in *z*-direction can be obtained by integrating all the *z*-direction projection of forces on spike/ACE2 in the contact zone, $F_Z = \int_{A} n \cdot f_{\varphi {{{{{{{\mathrm{Z}}}}}}}}}{{{{{{{\mathrm{d}}}}}}}}A$, which should be 0 for the equilibrium of the virion. Apparently, for any given *φ*_C_, the force in *z*-direction *F*_*Z*_ is a function of *R* and *l*_apex_. Therefore, *R* and *l*_apex_ are not independent. The selection of *R* and *l*_apex_ should lead to *F*_*Z=*_0 for the virion equilibrium.

The elastic potential energy of the virion and host-cell PM system consists of the PM bending energy and the spike/ACE2 bond elastic energy. During the virus entry, the host-cell PM can be divided into three parts: (1) the PM in contact with virus bending to a spherical surface; (2) the PM far away from the contact zone keeping in flat without bending; and (3) the PM in the transition zone joining the flat and spherical contact zone that bends to a surface with negative Gaussian curvature. The bending energy is zero in the flat membrane. To minimize the bending energy, the host-cell PM with negative Gaussian curvature must favor a minimal surface for two reasons: the surface area is minimal, and the tension is minimal; the mean curvature is zero, and the bending energy is zero. This minimal curvature membrane has also been found in virus–cell fusion.^78^ Therefore, we assume the host-cell PM in the transition zone approximately adopts a minimal surface, and the bending energy is dominantly stored in the spherical contact zone. It can be written as $E_{{{{{{{{\mathrm{mem}}}}}}}}} = \frac{1}{2} \cdot B\kappa ^2A = 4\pi B\left( {1 - \cos \varphi^{\prime} } \right)$, where κ is the mean curvature of the bent host-cell PM, 2/*R*, *A* is the contact zone area, and *B* (= 1.8 × 10^–19^ J)^79^ is bending modulus of the cell membrane. The elastic energy stored in each bond can be calculated by $E_\varphi = 1/2 \cdot k_{{{{{{{{\mathrm{mol}}}}}}}}} \cdot {\Delta} l^2$. The total elastic energy of all bonds is the integration of each individual one, $E_{{{{{{{{\mathrm{bond}}}}}}}}} = \int_{A} E_\varphi {{{{{{{\mathrm{d}}}}}}}}A = \int_{0}^{\varphi _{{{{{{{\mathrm{C}}}}}}}}} n \cdot \frac{1}{2}k_{{{{{{{{\mathrm{mol}}}}}}}}} \cdot \left( {l_\varphi - l_0} \right)^2 \cdot 2\pi r^2\sin \varphi d\varphi$. The total elastic potential energy is the sum of the aforementioned two parts: $E_{{{{{{{{\mathrm{tot}}}}}}}}} = E_{{{{{{{{\mathrm{mem}}}}}}}}} + E_{{{{{{{{\mathrm{bond}}}}}}}}}$. According to the theorem of minimum potential energy, the host-cell PM bending and spike/ACE2 bond deformation should reach the lowest total elastic potential energy. All this leads to a mathematic problem, that is, finding a displacement field of the host-cell PM for the minimum value of *E*_tot_ under the constrain of *F*_Z_ = 0. By using the sequential least squares programming algorithm, we could solve this problem at any given contact zone size *φ*_C_ (Fig. 1a, b).

### Force-dependent disassociation and S1/S2 detachment model of SARS2-S

The SARS2-S/ACE2 dissociation rate (*k*_off_) from ACE2 is the reciprocal of the average lifetime. By fitting the lifetime data with a logarithm and an exponential function for the catch (ascending) and slip (descending) phase respectively, an approximation relation between the force and dissociation rates was obtained. The SARS2-S can either unfold or dissociate from ACE2 first; if *k*_u_ > *k*_off_, S1/S2 is more likely to be detached before dissociation from ACE2. The probability of S1/S2 detachment first can be calculated by *k*_u_/(*k*_u_ + *k*_off_).

## Supplementary information

Supplementary information, Fig. S1

Supplementary information, Fig. S2

Supplementary information, Fig. S3

Supplementary information, Fig. S4

Supplementary information, Fig. S5

Supplementary information, Fig. S6

Supplementary information, Fig. S7

Supplementary information, Fig. S8

Supplementary information, Fig. S9

Supplementary information, Table S1

Supplementary information, Video S1

Supplementary information, Video S2

Supplementary information, Video S3

Supplementary information, Video S4

Supplementary information, Video S5

Supplementary information, Video Legend
