# Supplementary material for: Mechanical activation of spike fosters SARS-CoV-2 viral infection
Source: Cell Res. 2021 Aug 31;31(10):1047–60. doi: 10.1038/s41422-021-00558-x (PMC8406658; doi:10.1038/s41422-021-00558-x)
Supplement: Supplementary file 1 — Supplementary information, Fig. S1 [file 41422_2021_558_MOESM1_ESM.pdf]

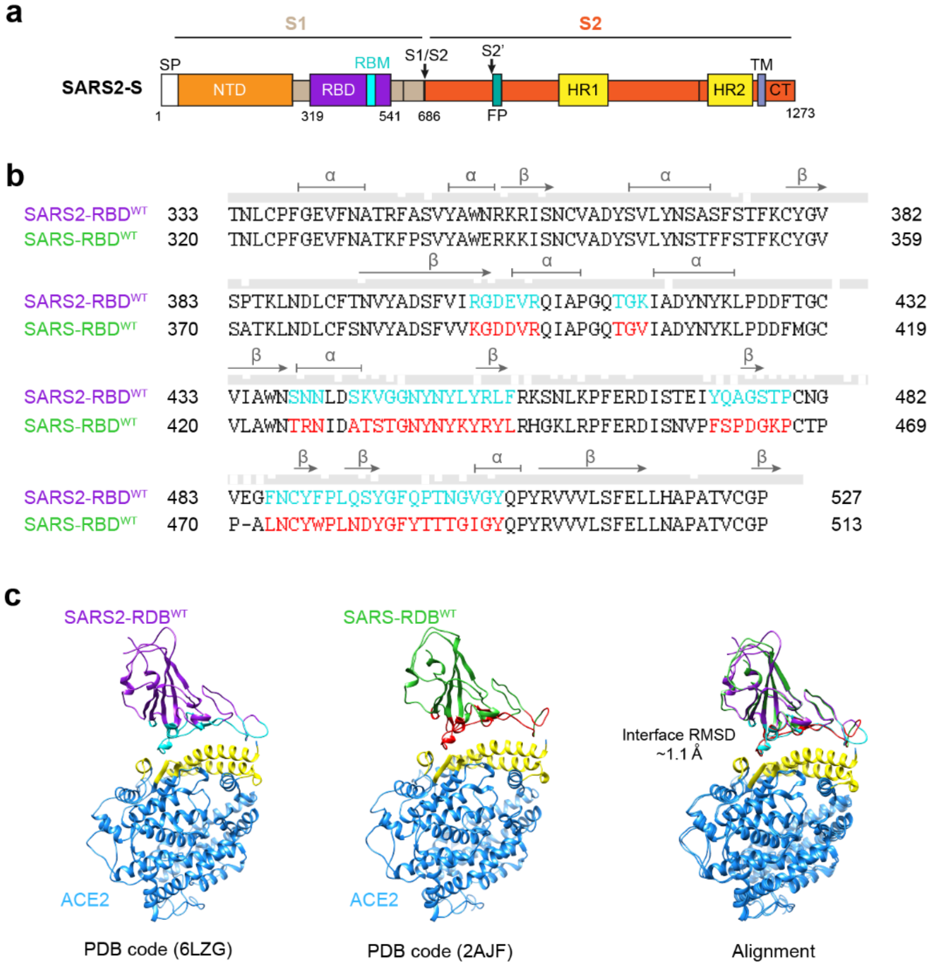

Fig. S1 The primary structure of SARS2-S and overall structures or sequences alignment of SARS2-RBD^{WT} and SARS-RBD^{WT} with ACE2.

a Schematic of SARS2-S primary structure. SP, NTD, RBD, RBM, FP, HR1, HR2, TM and CT, refer to signal peptide, N-terminal domain, receptor binding domain, receptor binding motif, fusion peptide, heptad repeat 1, heptad repeat 2, transmembrane domain and cytoplasmic tail, respectively. Arrows denote S1/S2 and S2' protease cleavage sites.

b Sequence and secondary structure arrangement of SARS2-RBD^{WT} and SARS-RBD^{WT}. The residues composed of the binding interface with ACE2 on SARS2-RBD^{WT} and SARS-RBD^{WT} are marked by cyan and red, respectively.

c Overall structure comparison of SARS2-RBD^{WT}/ACE2 (PDB: 6LZG) and SARS-RBD^{WT}/ACE2 (PDB: 2AJF) complexes. The residues composed of the interaction interface in SARS2-RBD^{WT} or SARS-RBD^{WT}/ACE2 are shown in cyan, red and yellow, respectively. Backbone RMSD of the interface between these two structures is ~1.1 Å.
