# Supplementary material for: Mechanical activation of spike fosters SARS-CoV-2 viral infection
Source: Cell Res. 2021 Aug 31;31(10):1047–60. doi: 10.1038/s41422-021-00558-x (PMC8406658; doi:10.1038/s41422-021-00558-x)
Supplement: Supplementary file 2 — Supplementary information, Fig. S2 [file 41422_2021_558_MOESM2_ESM.pdf]

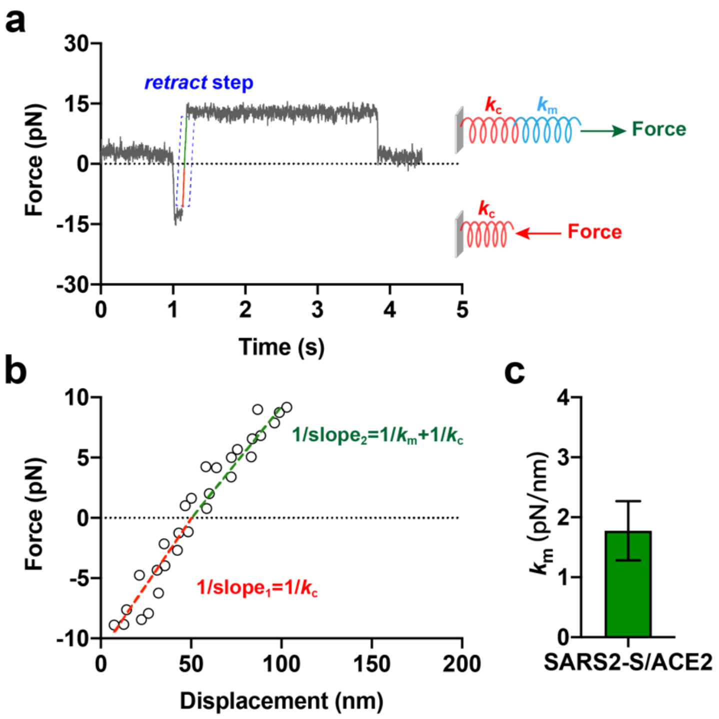

Fig. S2 Molecular stiffness analysis of single SARS2-S/ACE2 bond.

a Force *vs*. time trace of a representative BFP cycle from which molecular stiffness values were measured in *retract* step.

b Force *vs*. displacement trace to calculate molecular spring constant k_m, which was obtained from $1/slope_1=1/k_c$ and $1/slope_2=1/k_c+1/k_m$. $1/slope_1$ and $1/slope_2$ were evaluated by linear fit (red and blue dashed lines, respectively).

c The spring constant of spike/ACE2 bond was plotted with column bar.
