# Supplementary material for: Mechanical activation of spike fosters SARS-CoV-2 viral infection
Source: Cell Res. 2021 Aug 31;31(10):1047–60. doi: 10.1038/s41422-021-00558-x (PMC8406658; doi:10.1038/s41422-021-00558-x)
Supplement: Supplementary file 3 — Supplementary information, Fig. S3 [file 41422_2021_558_MOESM3_ESM.pdf]

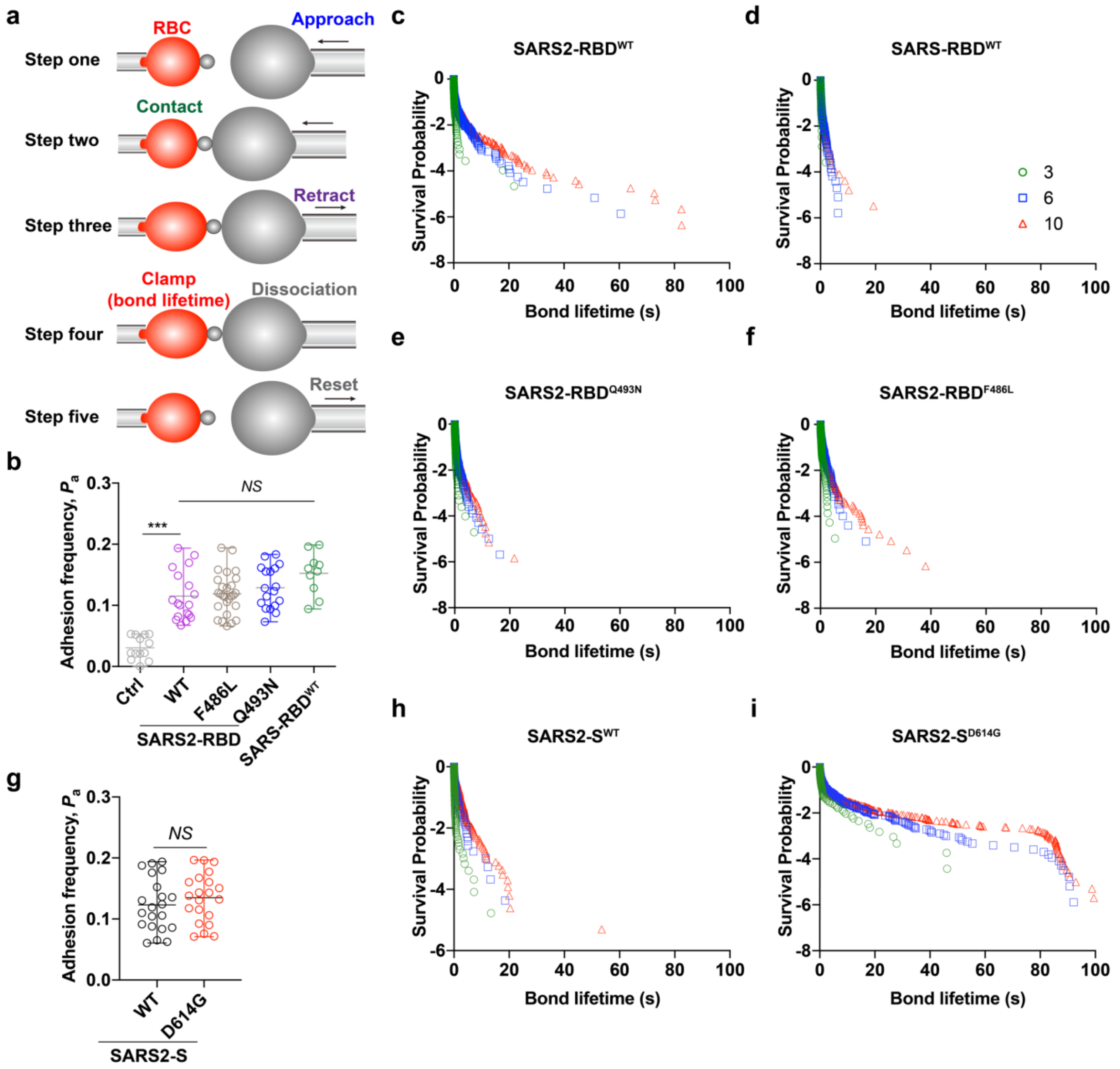

Fig. S3 BFP measurement and bond lifetimes analysis of the SARS2-RBD^{WT} or mutants, SARS-RBD^{WT}, SARS2-S^{WT} and SARS2-S^{D614G} with ACE2.

a Bond lifetime is determined through BFP-based force-clamp assay, which consists of five steps in one cycle (as indicated): approach, contact, retract, clamp and reset. During the 'clamp' process, the bond lifetime is collected.

b Single-molecule bond lifetime detection is guaranteed by adjusting adhesion frequency lower than 20% in >500 contacts. All error bars represent range. ***$p<0.001$, *NS* refers to no significant difference.

c-f Survival probabilities in single-bond lifetime measurements of ACE2 with SARS2-RBD^{WT} (c), SARS-RBD^{WT} (d) and SARS-RBD mutants (Q493N (e) and F486L (f)) under 3, 6 and 10 pN force in

the regime where force prolongs bond lifetimes.

g Single-molecule bond lifetime detection is guaranteed by adjusting adhesion frequency lower than 20% in >500 contacts. Both error bars represent range. *NS* refers to no significant difference.

h and i Survival probabilities in single-bond lifetime measurements of ACE2 with SARS2-S^{WT} (h) and SARS-S^{D614G} (i) under 3, 6 and 10 pN force in the regime where force prolongs bond lifetimes.
