# Supplementary material for: Mechanical activation of spike fosters SARS-CoV-2 viral infection
Source: Cell Res. 2021 Aug 31;31(10):1047–60. doi: 10.1038/s41422-021-00558-x (PMC8406658; doi:10.1038/s41422-021-00558-x)
Supplement: Supplementary file 4 — Supplementary information, Fig. S4 [file 41422_2021_558_MOESM4_ESM.pdf]

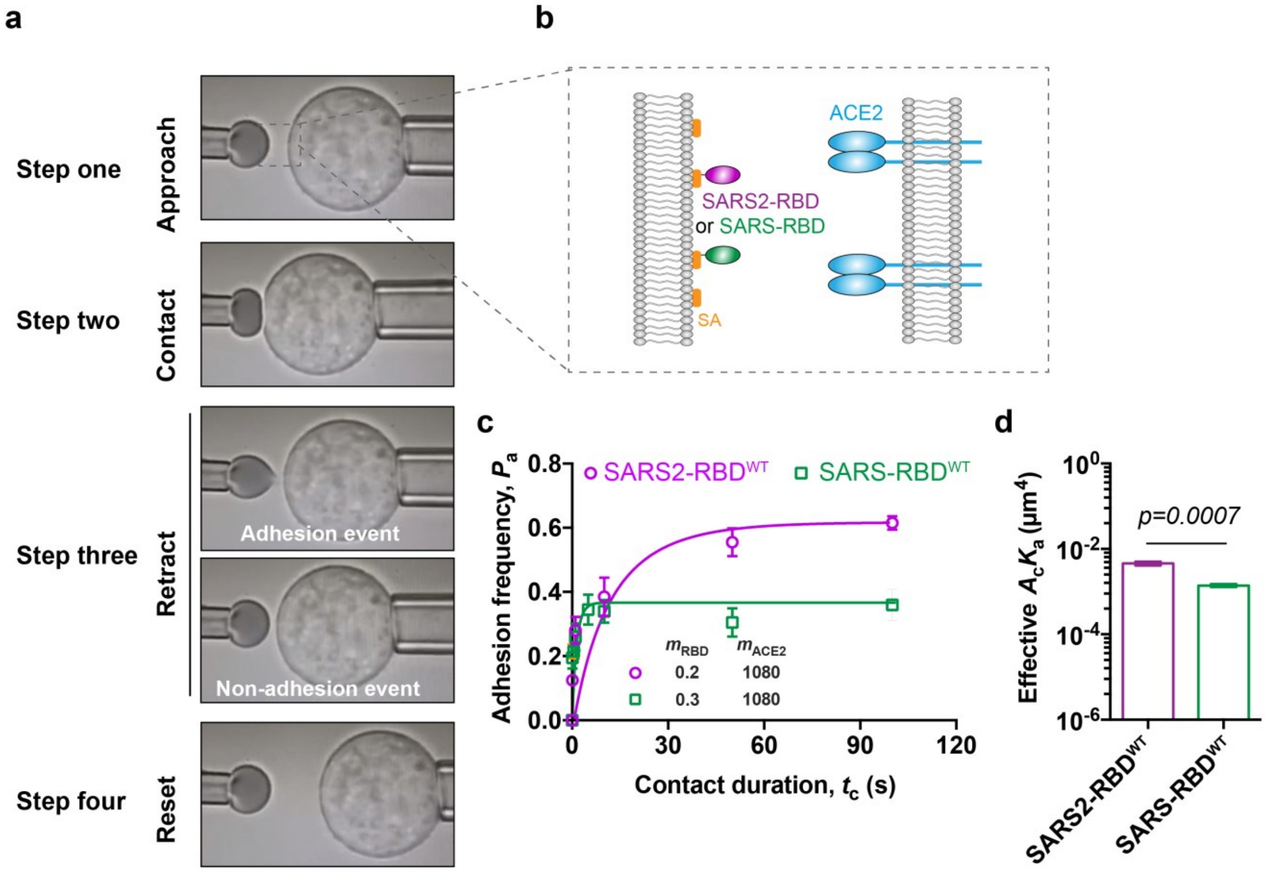

Fig. S4 SARS2-RBD^{WT} recognizes ACE2 with slightly higher binding affinity than SARS-RBD^{WT}.

a Representative micrographs of adhesion frequency assay, which consists of four steps in one cycle (as indicated): approach, contact, retract and reset. During the process from 'contact' to 'retract', adhesion event occurrence is judged by the deformation of RBC membrane.

b Functionalization of the adhesion frequency assay. RBC is coated with SARS2-RBD^{WT} or SARS-RBD^{WT} through streptavidin (SA)-biotin interaction and ACE2 is expressed on U937 cell.

c and d Adhesion frequency curves (c) and their derived in-situ 2D effective force-free binding affinity A_cK_a (d) of ACE2 with SARS2-RBD^{WT} (purple open circle) or SARS-RBD^{WT} (green open square). Molecular surface densities (m, μm^{-2}) of ACE2 and SARS2-RBD^{WT} or SARS-RBD^{WT} are indicated. All error bars represent SEM.
