# Supplementary material for: Mechanical activation of spike fosters SARS-CoV-2 viral infection
Source: Cell Res. 2021 Aug 31;31(10):1047–60. doi: 10.1038/s41422-021-00558-x (PMC8406658; doi:10.1038/s41422-021-00558-x)
Supplement: Supplementary file 5 — Supplementary information, Fig. S5 [file 41422_2021_558_MOESM5_ESM.pdf]

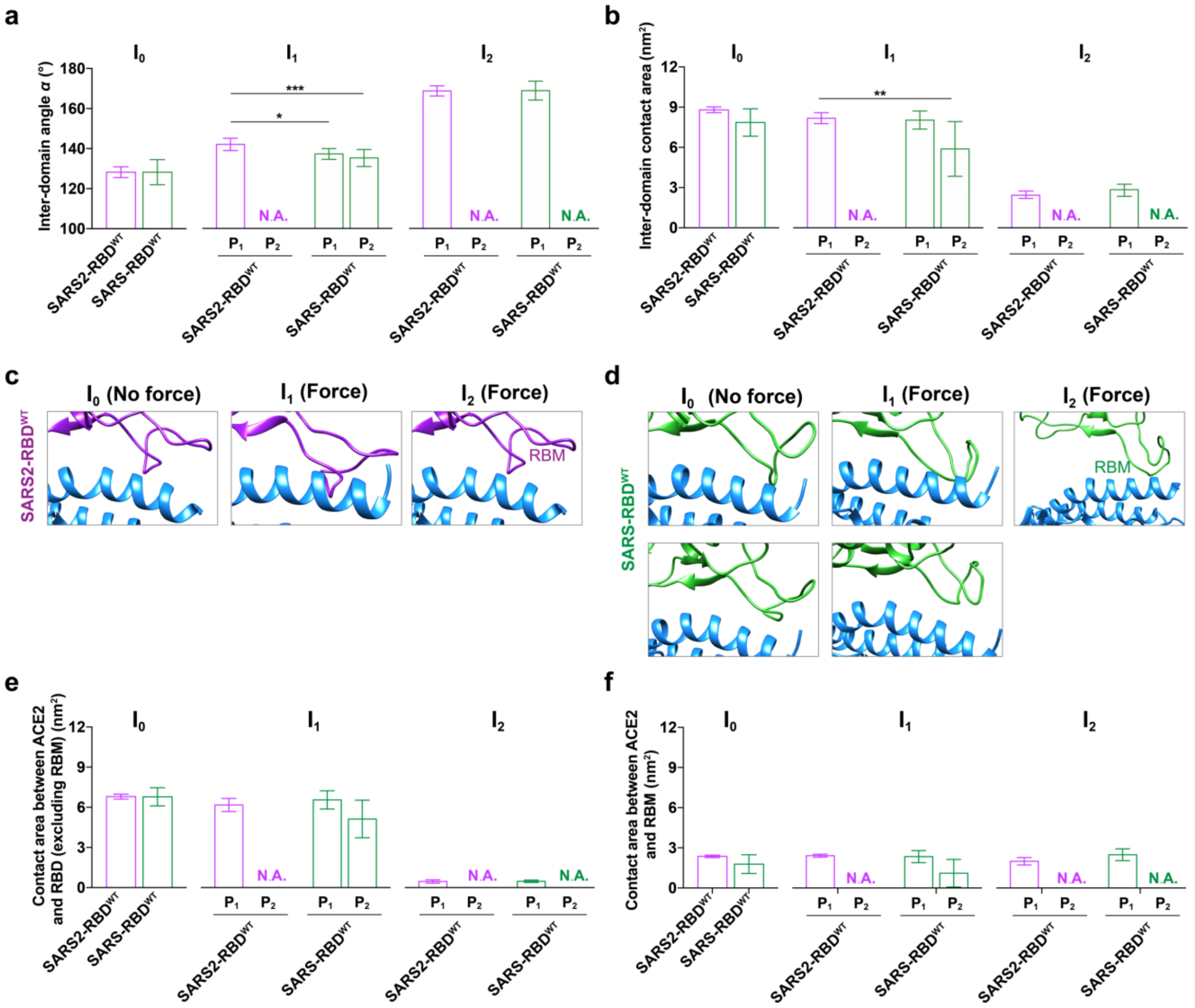

Fig. S5 Analysis of dynamic binding of RBD with ACE2 in SMD simulations.

a and b Comparisons of inter-domain angle (a) and contact area (b) in different dissociation pathways of SARS2-RBD^{WT} or SARS-RBD^{WT} with ACE2 and in different intermediate states (I_0, I_1 and I_2).

c and d Representative binding interface between RBM of SARS2-RBD^{WT} (c) or SARS-RBD^{WT} (d) and ACE2 in different intermediate states as indicated.

e The contact area between SARS2-RBD^{WT} or SARS-RBD^{WT} excluding RBM and ACE2 in different conformation states and dissociation pathways.

f The contact area between RBM of SARS2-RBD^{WT} or SARS-RBD^{WT} and ACE2 in different conformations states and dissociation pathways. All error bars represent SEM. *0.01<p<0.05, **0.001<p<0.01 and ***p<0.001. N.A. refers to not available.
