# Supplementary material for: Mechanical activation of spike fosters SARS-CoV-2 viral infection
Source: Cell Res. 2021 Aug 31;31(10):1047–60. doi: 10.1038/s41422-021-00558-x (PMC8406658; doi:10.1038/s41422-021-00558-x)
Supplement: Supplementary file 6 — Supplementary information, Fig. S6 [file 41422_2021_558_MOESM6_ESM.pdf]

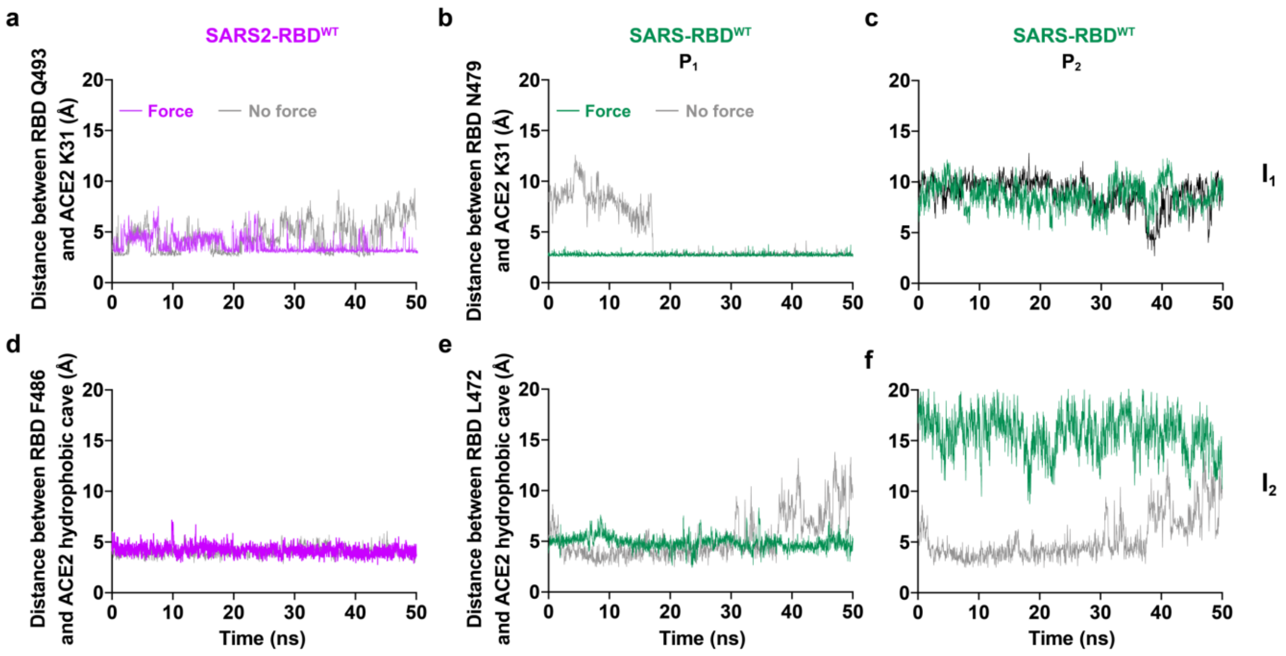

Fig. S6 Representative time-courses of the distances between paired residues in absence and presence of force.

a The time-courses of distances between Q493 of SARS2-RBD^{WT} and K31 of ACE2 in the force-free state (grey) and force-induced dissociation process (purple).

b and c The time-courses of distances between N479 of SARS-RBD^{WT} and K31 of ACE2 in force-free state (grey) and force-induced dissociation process along with P_1 (b) or P_2 (c) pathway (green).

d The time-courses of distances between F486 of SARS2-RBD^{WT} and ACE2 hydrophobic center composed by residues L79, M82 and Y83 in the force-free state (grey) and force-induced dissociation process (purple).

e and f The time-courses of distances between L472 of SARS-RBD^{WT} and ACE2 hydrophobic center composed by residues L79, M82 and Y83 in force-free state (grey) and force-induced dissociation process along with P_1 (e) or P_2 (f) pathway (green).
