# Supplementary material for: Mechanical activation of spike fosters SARS-CoV-2 viral infection
Source: Cell Res. 2021 Aug 31;31(10):1047–60. doi: 10.1038/s41422-021-00558-x (PMC8406658; doi:10.1038/s41422-021-00558-x)
Supplement: Supplementary file 7 — Supplementary information, Fig. S7 [file 41422_2021_558_MOESM7_ESM.pdf]

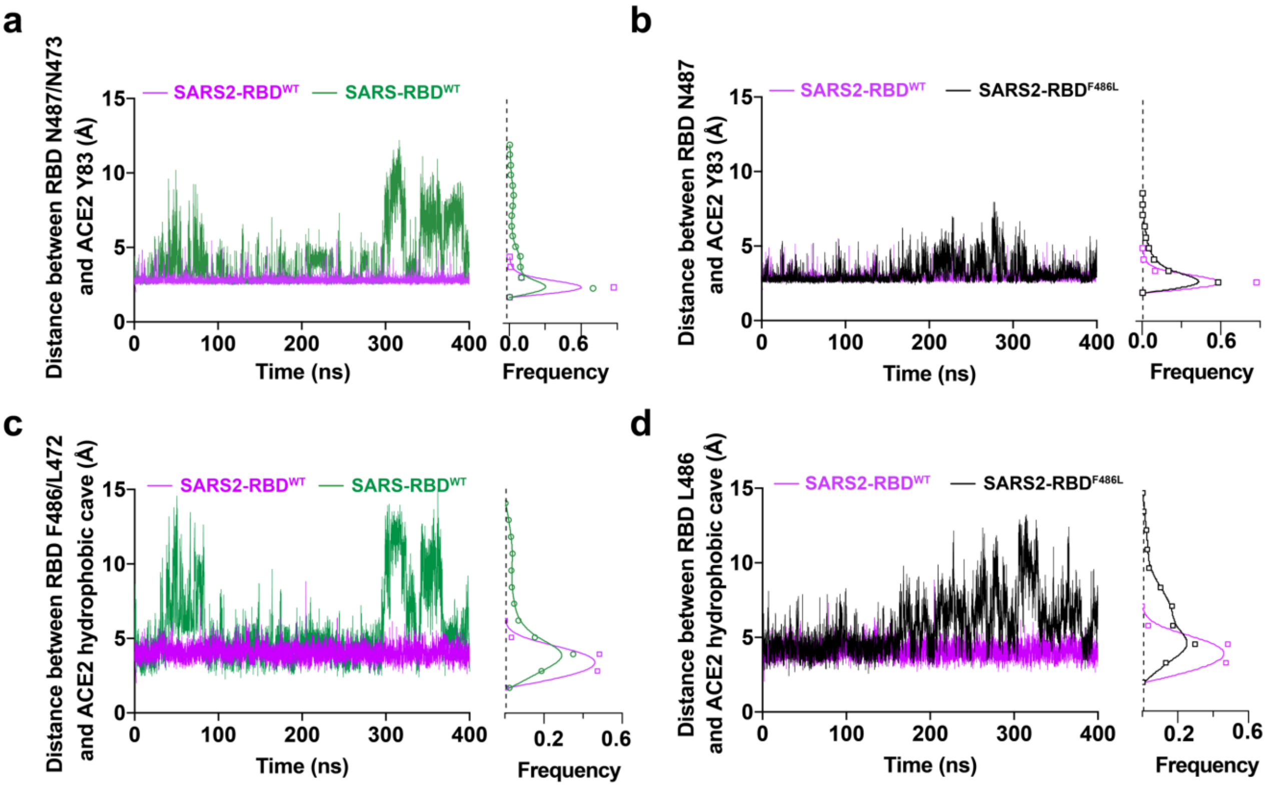

Fig. S7 Time-dependent interactions between paired residues in SMD simulations.

a-d The time-courses and distributions of the distances between N487 of SARS2-RBD^{WT} or N473 of SARS-RBD^{WT} and Y83 of ACE2 (a), N487 of SARS2-RBD^{F486L} and Y83 of ACE2 (b), F486 of SARS2-RBD^{WT} or L472 of SARS-RBD^{WT} and ACE2 hydrophobic center composed by L79, M82 and Y83 (c), or L486 of SARS2-RBD^{F486L} and ACE2 hydrophobic center composed by L79, M82 and Y83 (d).
