# Supplementary material for: Mechanical activation of spike fosters SARS-CoV-2 viral infection
Source: Cell Res. 2021 Aug 31;31(10):1047–60. doi: 10.1038/s41422-021-00558-x (PMC8406658; doi:10.1038/s41422-021-00558-x)
Supplement: Supplementary file 8 — Supplementary information, Fig. S8 [file 41422_2021_558_MOESM8_ESM.pdf]

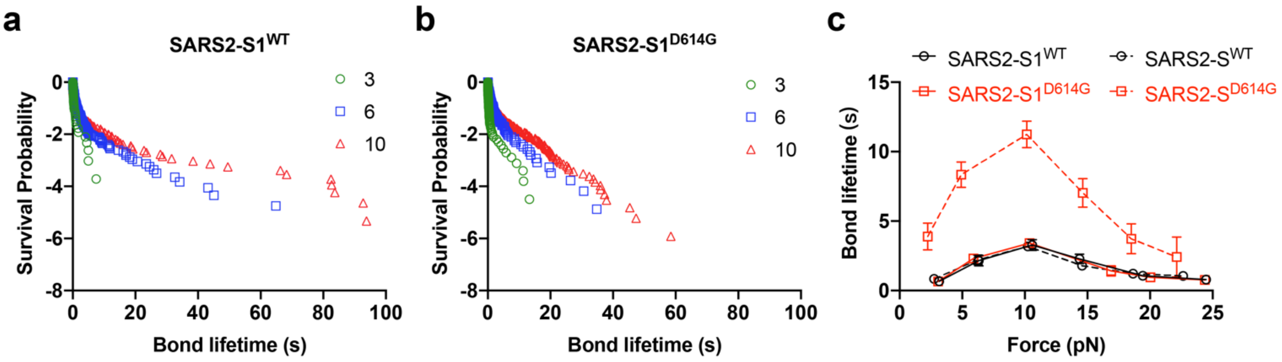

Fig. S8 BFP measurement and bond lifetimes analysis of the SARS2-S1^{WT} and SARS2-S1^{D614G} monomer with ACE2.

a and b Survival probabilities in single-bond lifetime measurements of ACE2 with SARS2-S1^{WT} (a) and SARS2-S1^{D614G} under 3, 6 and 10 pN force in the regime where force prolongs bond lifetimes.

c Force-dependent bond lifetimes of SARS2-S1^{WT} (black solid plots) or SARS2-S1^{D614G} (red solid plots) monomer binding with ACE2, in comparison with SARS2-S^{WT} (black dashed plots) or SARS2-S^{D614G} (red dashed plots) trimer. Error bars represent SEM.
