# Supplementary material for: Mechanical activation of spike fosters SARS-CoV-2 viral infection
Source: Cell Res. 2021 Aug 31;31(10):1047–60. doi: 10.1038/s41422-021-00558-x (PMC8406658; doi:10.1038/s41422-021-00558-x)
Supplement: Supplementary file 9 — Supplementary information, Fig. S9 [file 41422_2021_558_MOESM9_ESM.pdf]

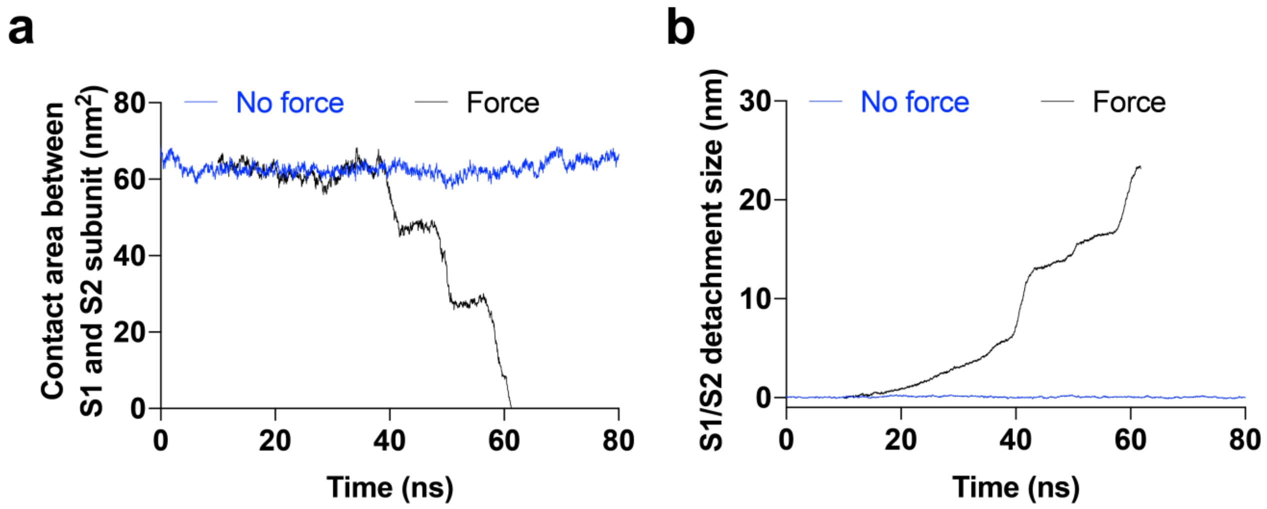

Fig. S9 SARS2-S S1/S2 detachment is observed under force through SMD.

a and b Time-courses of contact area (a) between S1 and S2 and their detachment size of SARS2-S^{WT} in presence (black line) and absence (blue line) of force (b), which are revealed by SMD.
