# Supplementary material for: Mechanical activation of spike fosters SARS-CoV-2 viral infection
Source: Cell Res. 2021 Aug 31;31(10):1047–60. doi: 10.1038/s41422-021-00558-x (PMC8406658; doi:10.1038/s41422-021-00558-x)
Supplement: Supplementary file 10 — Supplementary information, Table S1 [file 41422_2021_558_MOESM10_ESM.pdf]

Table S1. The kinetics and binding affinities of SARS2 and ACE2 interactions.

SARS2	Adhesion frequency assay					Biolayer interferometry binding assay		
	m_{SARS2} (μm^{-2})	m_{ACE2} (μm^{-2})	A_cK_a (×10^{-3} μm^4)	A_ck_{on} (×10^{-4} μm^4 s^{-1})	k_{off} (×10^{-2} s^{-1})	K_D (nM)	k_{on} (×10^5 M^{-1} s^{-1})	k_{off} (×10^{-2} s^{-1})
RBD^{WT}	0.2	1080	4.57±0.49	2.60±0.28	5.69±2.315	66.71±0.59	1.81±0.01	1.21±0.01
RBD^{Q493N}	0.3	1080	3.03±0.35	5.53±0.64	18.22±7.721	88.6±1.16	2.04±0.02	1.81±0.01
RBD^{F486L}	0.3	1080	2.45±0.28	3.11±0.36	12.68±6.221	124.90±2.29	1.72±0.03	2.14±0.01
S^{WT}	0.2	1080	6.75±0.63	6.15±0.58	9.11±1.63			
S^{D614G}	0.5	1080	2.35±0.24	2.41±0.24	10.25±2.24			
