# Supplementary material for: Mechanical activation of spike fosters SARS-CoV-2 viral infection
Source: Cell Res. 2021 Aug 31;31(10):1047–60. doi: 10.1038/s41422-021-00558-x (PMC8406658; doi:10.1038/s41422-021-00558-x)
Supplement: Supplementary file 16 — Supplementary information, Video Legend [file 41422_2021_558_MOESM16_ESM.pdf]

Video S1: The host-cell plasma membrane bent to adopt the virion shape once viral attachment by spike/ACE2 interaction (left), and the theoretical estimation of the pulling (red) and compressing (blue) force exerted on single spike/ACE2 bond when the contact zone grows (right).

Video S2: Representative SMD movie of force-dependent SARS2-RBD^{WT} (purple) dissociation from ACE2 (cyan) along sole P_1 pathways. The key residues, F486 and Q493 of SARS2-RBD^{WT} and K31, L79, M82 and Y83 of ACE2, are shown. The complex structures are orientated by aligning ACE2-PD.

Video S3: Representative SMD movie of force-dependent SARS-RBD^{WT} (lime) dissociation from ACE2 (cyan) along P_1 pathways. The key residues, L472 and N479 of SARS-RBD^{WT} and K31, L79, M82 and Y83 of ACE2, are shown. The complex structures are orientated by aligning ACE2-PD.

Video S4: Representative SMD movie of force-dependent SARS-RBD^{WT} (lime) dissociation from ACE2 (cyan) along P_2 pathways. The key residues, L472 and N479 of SARS-RBD^{WT} and K31, L79, M82 and Y83 of ACE2, are shown. The complex structures are orientated by aligning ACE2-PD.

Video S5: Representative SMD movie of force-dependent SARS2-S^{WT} S1/S2 detachment. NTD (orange), RBD (purple) and S2 (green) subunits of one protomer are colored and other two protomers labeled with gray.
